# Hunting and mountain sheep: Do current harvest practices affect horn growth?

**DOI:** 10.1111/eva.12841

**Published:** 2019-07-29

**Authors:** Tayler N. LaSharr, Ryan A. Long, James R. Heffelfinger, Vernon C. Bleich, Paul R. Krausman, R. Terry Bowyer, Justin M. Shannon, Robert W. Klaver, Clay E. Brewer, Mike Cox, A. Andrew Holland, Anne Hubbs, Chadwick P. Lehman, Jonathan D. Muir, Bruce Sterling, Kevin L. Monteith

**Affiliations:** ^1^ Wyoming Cooperative Fish and Wildlife Research Unit, Department of Zoology and Physiology University of Wyoming Laramie WY USA; ^2^ Department of Fish and Wildlife Sciences University of Idaho Moscow ID USA; ^3^ Arizona Game and Fish Department Phoenix AZ USA; ^4^ Department of Natural Resources and Environmental Science University of Nevada Reno Reno NV USA; ^5^ School of Natural Resources and the Environment University of Arizona Tucson AZ USA; ^6^ Institute of Arctic Biology University of Alaska Fairbanks Fairbanks AK USA; ^7^ Utah Division of Wildlife Resources Salt Lake City UT USA; ^8^ US Geological Survey, Iowa Cooperative Fish and Wildlife Research Unit, Department of Natural Resource Ecology and Management Iowa State University Ames IA USA; ^9^ Western Association of Fish and Wildlife Agencies—Wild Sheep Working Group Texas Parks and Wildlife Department Rochelle TX USA; ^10^ Nevada Department of Wildlife Reno NV USA; ^11^ Colorado Parks and Wildlife Fort Collins CO USA; ^12^ Alberta Environment and Parks Rocky Mountain House AB Canada; ^13^ South Dakota Game Fish and Parks Custer SD USA; ^14^ Oregon Department of Fish and Wildlife Lakeview OR USA; ^15^ Montana Fish, Wildlife, and Parks Thompson Falls MT USA; ^16^ Haub School of Environment and Natural Resources, Wyoming Cooperative Fish and Wildlife Research Unit, Department of Zoology and Physiology University of Wyoming Laramie WY USA

**Keywords:** artificial evolution, bighorn sheep, harvest‐induced evolution, horns, selective harvest, trophy hunting

## Abstract

The influence of human harvest on evolution of secondary sexual characteristics has implications for sustainable management of wildlife populations. The phenotypic consequences of selectively removing males with large horns or antlers from ungulate populations have been a topic of heightened concern in recent years. Harvest can affect size of horn‐like structures in two ways: (a) shifting age structure toward younger age classes, which can reduce the mean size of horn‐like structures, or (b) selecting against genes that produce large, fast‐growing males. We evaluated effects of age, climatic and forage conditions, and metrics of harvest on horn size and growth of mountain sheep (*Ovis canadensis* ssp.) in 72 hunt areas across North America from 1981 to 2016. In 50% of hunt areas, changes in mean horn size during the study period were related to changes in age structure of harvested sheep. Environmental conditions explained directional changes in horn growth in 28% of hunt areas, 7% of which did not exhibit change before accounting for effects of the environment. After accounting for age and environment, horn size of mountain sheep was stable or increasing in the majority (~78%) of hunt areas. Age‐specific horn size declined in 44% of hunt areas where harvest was regulated solely by morphological criteria, which supports the notion that harvest practices that are simultaneously selective and intensive might lead to changes in horn growth. Nevertheless, phenotypic consequences are not a foregone conclusion in the face of selective harvest; over half of the hunt areas with highly selective and intensive harvest did not exhibit age‐specific declines in horn size. Our results demonstrate that while harvest regimes are an important consideration, horn growth of harvested male mountain sheep has remained largely stable, indicating that changes in horn growth patterns are an unlikely consequence of harvest across most of North America.

## 1 INTRODUCTION

Understanding the ecological and evolutionary responses of wild populations to anthropogenic change is important for the management and conservation of wildlife. Human activities around the globe have led to increased global temperatures (Deutsch et al., 2008; Parmesan, Singer, & Harris, 1995), fragmented and degraded habitats (Fahrig, 2003; Ferraz et al., 2007), and pollution (Butchart, 2010; Verhoeven, Arheimer, Yin, & Hefting, 2006). In addition, several recent studies have suggested that harvest by humans can cause evolutionary changes in some populations (Allendorf & Hard, 2009). Harvest‐induced evolution has important implications for management and persistence of many wild species across the world (Allendorf, England, Luikart, Ritchie, & Ryman, 2008; Kuparinen & Festa‐Bianchet, 2017); yet, potential evolutionary effects of harvest on wild populations rarely have been studied at temporal scales sufficient to detect evolutionary change, especially for long‐lived species (Corlatti, Storch, Filli, & Anderwald, 2017; Hundertmark, Thelen, & Bowyer, 1998). Even at limited temporal and spatial scales, however, evolutionary responses to harvest have been documented in several taxa (Allendorf et al., 2008; Coltman et al., 2003; Walsh, Munch, Chiba, & Conover, 2006). Populations that are subjected to sufficiently intensive and selective harvest may exhibit reduced horn or antler size, reduced growth rate, early sexual maturation, altered behaviors (e.g., foraging, courtship, and migration behaviors), and changes to life‐history strategies over only a few generations (Allendorf & Hard, 2009; Darimont, Fox, Bryan, & Reimchen, 2015; Devine, Wright, Pardoe, Heino, & Fraser, 2012; Hard et al., 2008; Monteith et al., 2013; Olsen, Heupel, Simpfendorfer, & Moland, 2012; Walsh et al., 2006).

The threshold of harvest necessary to produce such evolutionary changes remains unclear for most species. One reason is that evolutionary changes resulting from harvest often mimic changes caused by phenotypic plasticity in response to variation in environmental conditions (Kuparinen & Festa‐Bianchet, 2017) or density‐dependent processes (Bowyer, Bleich, Stewart, Whiting, & Monteith, 2014). Consequently, disentangling the relative strength of selection imposed by harvest versus effects caused by environmental conditions is a challenging endeavor. Nevertheless, meeting this challenge is imperative for understanding how and to what degree harvest‐induced evolution is occurring. For example, in fishes, declining population density because of harvest can increase per capita resource availability, leading to accelerated juvenile growth and early sexual maturation, which can result in small body size at sexual maturity (Kuparinen & Merilä, 2007; Sinclair, Swain, & Hanson, 2002). Such changes are similar to those expected from harvest‐induced evolution (Walsh et al., 2006), and yet the underlying mechanisms, as well as the potential implications for management, are quite different. In contrast, increasing population density of ungulates reduces per capita availability of forage, which can result in a shift in allocation of resources (i.e., energy and protein) from growth of secondary sexual characteristics (i.e., horns and antlers, referred to from here on as weapons) to growth and maintenance of somatic tissue (Monteith et al., 2018). Plastic shifts in resource allocation in response to limited availability of those resources can produce negative temporal trends in the size of weapons that mimic trends expected to arise from harvest‐induced evolution (Festa‐Bianchet, 2017).

Weapon size of large ungulates is a heritable trait (Kruuk et al., 2002; Pigeon, Festa‐Bianchet, Coltman, & Pelletier, 2016) that plays a role in reproductive success through male–male combat (Bubenik & Bubenik, 1990; Goss, 1983), and can be an important determinant of fitness (Poissant, Wilson, Festa‐Bianchet, Hogg, & Coltman, 2008). The size of weaponry is influenced by genetics (Kruuk et al., 2002), but also is dependent upon the resources necessary for growth. As a result, weapon size is thought to be an indicator of individual quality (Malo, Roldan, Garde, Soler, & Gomendio, 2005; Vanpe et al., 2007). In addition to the biological significance of ungulate weaponry, there is substantial cultural and sociological interest in such weaponry among humans. Weapon size of harvested animals is highly valued by an increasingly “hornographic” culture wherein the desire to harvest a specimen with exceptionally large weaponry is notable (Heffelfinger, 2018; Monteith et al., 2018).

Ungulate species can exhibit accelerated changes in weapon size in response to the selective removal of individuals with large weaponry (Festa‐Bianchet, Jorgenson, & Réale, 2000; Hard & Mills, 2006; Monteith et al., 2013; Pigeon et al., 2016). Nevertheless, it remains challenging to disentangle the effects of natural processes from selective pressures of harvest, especially given that long‐term data on phenotypic traits are exceedingly rare (Festa‐Bianchet & Mysterud, 2018; Hundertmark et al., 1998; LaSharr et al., 2019; Monteith et al., 2013). Although the level of harvest pressure necessary to produce evolutionary changes in the size of weaponry has been examined in a theoretical context (Festa‐Bianchet 2016; Mysterud, 2011), few empirical studies have directly tested the effects of harvest practices on weapon size. Despite the uncertainty that still surrounds the effects of harvest on weapon size of ungulates, a growing body of popular literature continues to suggest that the practice of hunting males with large horn‐like structures results in “reverse evolution” or can drive species toward extinction (Britt, 2009; Gabbatiss, 2017; Huang, 2009; Leahy, 2017). Consequently, there has been increased concern among the public about the general sustainability of harvest practices across the world. Indeed, mountain sheep have been the focus of much of the controversy surrounding the evolutionary effects of harvest in terrestrial species (Boyce & Krausman, 2018) since the early 2000s. Nevertheless, a variety of confounding factors may reduce the effectiveness of selective harvest in producing a detectable evolutionary change to horn size; these factors are related primarily to the heritability of selected traits, genetic contribution of females, nutrition, gene flow, and gene linkage (see Heffelfinger (2018) for a review of these concerns). Further, intensive modeling efforts have indicated that evolutionary changes may occur so slowly that it could take tens of generations before a detectable change manifests (Coulson, Schindler, Traill, & Kendall, 2018; Mysterud & Bischof, 2010).

Under harvest regulated solely by a morphological criterion on Ram Mountain, Alberta, Canada, marked reductions in horn length of bighorn sheep over 26 years were explained partially by genetic effects of harvest (Pigeon et al., 2016). The change in horn length with associated genetic change yields empirical evidence that sufficiently selective and intensive harvest can result in an evolutionary change in potentially as few as five generations (Pigeon et al., 2016). It remains unclear, however, whether the management of mountain sheep throughout their native range should promote similar concerns to those that have been raised on Ram Mountain, and thus, harvest of mountain sheep remains a controversial topic among managers, biologists, and wildlife researchers. Identifying how harvest practices across mountain sheep range may influence horn growth has the potential to shed light on evolutionary consequences of selective harvest and the sustainability of current harvest regimes.

Mountain sheep are ideal for testing the effects of harvest on weapon size of ungulates. Harvest of mountain sheep throughout their range in the United States and Canada is closely monitored (Monteith et al., 2018), and successful hunters are required to have harvested specimens examined by the management agency responsible for the area where the animal was taken. Consequently, an incredible amount of information on phenotypic characteristics of mountain sheep has been collected through time as management agencies have recorded data on age and horn size of harvested specimens for multiple decades. Furthermore, the bulk of the current evidence supporting an effect of selective harvest on ungulate species has been obtained from studies of mountain sheep, where extensive pedigrees and assessments of phenotypic and genotypic changes in horn size have demonstrated that harvest can reduce the size of weaponry through time (Coltman, 2008; Pigeon et al., 2016). This evidence, however, largely has stemmed from a single population (Ram Mountain, Alberta, Canada) with unique characteristics and a harvest regime that is not employed in other jurisdictions across most of mountain sheep range, with the notable exception of management areas in most of Alberta, Canada. Ideally, assessing the effect of selective harvest on wild populations would include sophisticated molecular approaches (Coltman, 2008). Such data are not readily available, however, and conducting molecular analyses at the temporal and spatial scales necessary to encompass variation in harvest practices and evolutionary change in a long‐lived mammal is expensive.

We sought to evaluate how demographic changes, selective harvest, and environmental characteristics influenced horn size and growth of Rocky Mountain bighorn sheep (*Ovis canadensis canadensis)* and desert bighorn sheep (*Ovis canadensis nelsoni* and related subspecies) that were harvested across 9 U.S. states and 1 Canadian province between 1981 and 2016. Through a hypothesis‐driven, weight‐of‐evidence approach, we indirectly tested for the effects of selective harvest on horn growth by first accounting for other factors that influence size and growth of horns (e.g., age and the environment), and then assessing the influence of harvest intensity and selectivity on unexplained variation in horn growth through time. We tested three hypotheses associated with the effects of harvest and environment on temporal changes in horn size of mountain sheep throughout much of their North American range. We considered hypotheses to not be mutually exclusive, but instead acknowledge that each could be operating in a location simultaneously.

### 1.1 Demographic shift hypothesis (H1)

**Figure 1:**
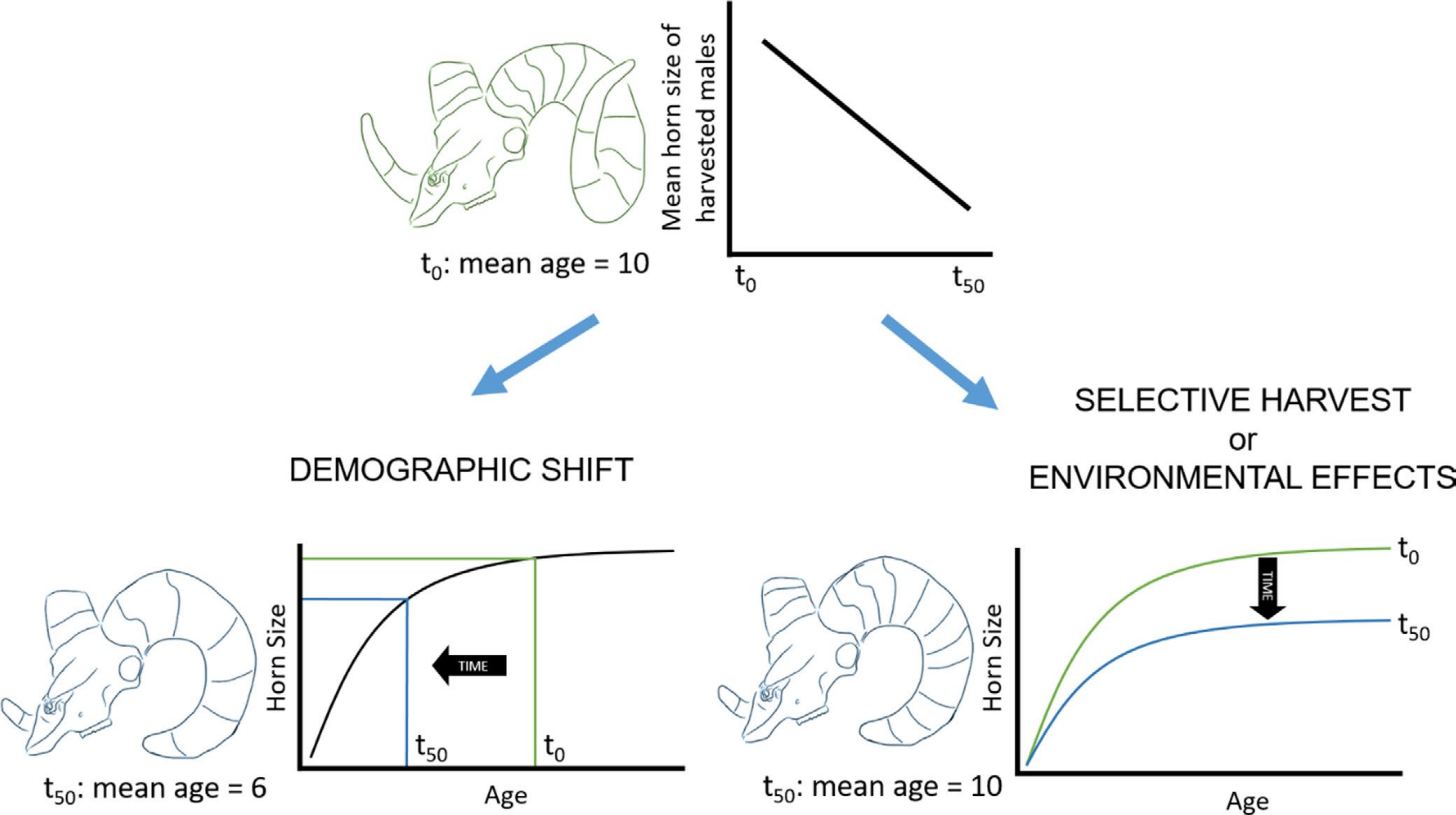
The mechanisms that can influence population‐level changes in horn size of mountain sheep through time. Population‐level changes in horn size can occur via two pathways. First, changes in demography that result in a declining age structure can reduce the mean age of harvested animals over time. Declining age of harvested animals produces a corresponding reduction in mean horn size. Alternatively, harvest selectivity and intensity or changes in environmental conditions can produce age‐specific changes in horn size through time that are independent of age structure. For example, highly selective and intensive harvest or poor environmental conditions may reduce horn growth through time, resulting in age‐specific declines in horn size

We assessed the hypothesis that changes in the age structure of a population through time would result in temporal changes in the mean horn size of individuals harvested from that population. Horn size is dependent on age (Bunnell, 1978; Geist, 1966), and we predicted a positive relationship between the proportion of older individuals harvested and mean horn size of harvested males (Figure 1).

Age is the most important determinant of horn size, but genetics and environmental conditions also have important implications for horn size and growth. Irrespective of changes in age structure, shifts in the horn growth curve (i.e., the relationship between age and horn size) of a population still can result from hunter selectivity for males with fast‐growing and large horns (Pigeon et al., 2016), or through variation in environmental conditions that influence nutritional condition and the allocation of resources to horn growth (Monteith et al., 2018; Monteith, Schmitz, Jenks, Delger, & Bowyer, 2009). In both instances, we would expect age‐specific changes in horn size through time.

### 1.2 Environmental effects hypothesis (H2)

We assessed the hypothesis that environmental conditions, namely indices of climate and forage availability, would influence horn growth through time (Figure 1). We predicted that harsh climatic conditions, poor forage availability, or both, would cause declines in age‐specific horn size, whereas mild climatic conditions, favorable forage conditions, or both, would increase age‐specific horn size (Geist, 1971). We analyzed the effects of environmental conditions on cohorts of animals at three temporal scales: in the year before a cohort was born, during the first 3 years of life, and throughout life. Conditions experienced by a dam during gestation can influence both body size and weapon size of her offspring throughout its life (Michel et al., 2016; Monteith et al., 2009), and because mountain sheep take several years to reach adult body size, environmental conditions during that developmental period may influence the trade‐off between allocation of resources to somatic tissue and growth of horns (Festa‐Bianchet, Coltman, Turelli, & Jorgenson, 2004; Geist, 1966; Robinson, Pilkington, Clutton‐Brock, Pemberton, & Kruuk, 2006). Finally, horns of mountain sheep grow continually throughout life and environmental conditions throughout an individual's life can have important influences on ultimate horn size (Monteith et al., 2013).

### 1.3 Selective harvest hypothesis (H3)

Finally, we evaluated the hypothesis that selective harvest of males with large and fast‐growing horns would result in an evolutionary change in horn size through time by favoring the survival and potential reproductive advantage incurred by males with small and slow‐growing horns. Sufficient removal of males with large and fast‐growing horns will favor the persistence of males with small and slow‐growing horns, which could result in an evolutionary change through time (Pigeon et al., 2016). After accounting for age and environmental conditions, we predicted that harvest pressure that was sufficiently intense and selective would produce age‐specific declines in horn size through time.

## 2 MATERIALS AND METHODS

We evaluated the effects of harvest, climate, and forage availability on horn size of mountain sheep using harvest records collected by state and provincial agencies from 1981 to 2016. We obtained harvest records for two subspecies of mountain sheep (Rocky Mountain bighorn sheep and desert bighorn sheep) from nine states in the United States and one Canadian province. Wildlife managers and biologists throughout the range of mountain sheep collected data on age and size of horns from harvested animals for decades. State and provincial agencies typically require hunters to have all harvested mountain sheep examined immediately after harvest, and age and horn measurements are recorded at that time. Those measurements represent one of the only datasets in North America for which age of the animal and a metric of horn size have been collected simultaneously for any ungulate across such broad spatial and temporal scales.

**Figure 2:**
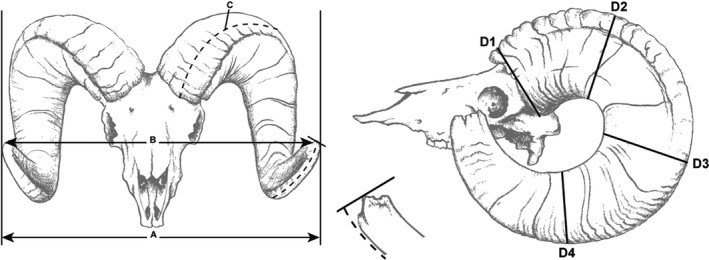
Illustrations of horn measurements for mountain sheep used by state and provincial agencies throughout western United States and Canada. Measurement criteria follow protocols established by the Boone and Crockett Record Book Program (Buckner & Reneau, 2009). The full score was calculated as the cumulative sum of C and all D measurements for both horns. The length–base score was calculated as the cumulative score of the C measurement for the longest horn twice, and the D1 measurement for both horns

We used two different metrics of horn size in our analyses because of differences in measurement data obtained from state or provincial agencies: (a) full score and (b) length–base score. Full score was calculated by summing the length of the outer edge of the horn and 4 circumference measurements equally spaced along each horn (Figure 2). Length–base score was calculated by doubling the length of the outer edge of the longest horn and adding that value to the measurements of the basal circumference of each horn (Figure 2). We used the measurement of the longest horn twice for the length–base score to reduce bias that may arise if one horn was broomed heavily (i.e., tips of horns were broken or worn off). We did not use the longest horn twice for the full score because agencies that provided us with full scores often did not have individual measurements available, and provided only the total score. Measurement protocols used by state and provincial agencies were based on the scoring system developed by the Boone and Crockett Club (Buckner & Reneau, 2009).

### 2.1 Weather and plant phenology

To evaluate the effects of climate and forage on horn growth through time, we extracted spatially explicit data on precipitation, snow water equivalent, and minimum temperature from 1981 to 2016 using modeled values from DAYMET (1‐km^2^ resolution) from 1 October to 31 May. Snow water equivalent, minimum temperature, and precipitation during winter are indicative of winter severity (Dawe & Boutin, 2012) and thus have implications for the nutritional condition of sheep and their associated ability to allocate resources to horn growth. Precipitation in desert systems influences water availability, and therefore condition, of desert bighorn sheep (Cain, Krausman, Morgart, Jansen, & Pepper, 2008; Gedir et al., 2016). We calculated mean values of each variable at three temporal scales for each individual sheep: year of gestation, the first 3 years of life, and the entire lifetime of the individual.

To assess the effects of forage availability and quality on sheep nutrition, and therefore horn size, we used version 3g.v1 NDVI (Normalized Difference Vegetation Index) obtained from the Global Inventory Monitoring and Modeling System (GIMMS, https://ecocast.arc.nasa.gov/data/pub/gimms/). These data were assembled from different AVHRR (Advanced Very High Resolution Radiometer) sensors and accounted for calibration loss, volcanic eruptions, radiometric calibration, atmospheric correction and cloud screening, and solar zenith angle correction (Tucker et al., 2005). The NDVI data were 15‐day composites in geographic coordinates with a WGS‐1984 map datum and a pixel size of 0.0833°; thus, there were 24 images per calendar year. We used the gimms package (Detsch, 2016) in Program R to download and rasterize those data for North America.

For each hunt area, we extracted NDVI values from herd ranges of mountain sheep that were identified by state and provincial agencies as occupied habitat (data provided by the Wild Sheep Working Group). We used the extract function in the raster package of Program R (Hijmans, 2017) to calculate the spatial mean of NDVI in each sheep range during each 15‐day period. We then created a time series of those spatial means during 1982–2015 to calculate phenology metrics with TIMESAT 3.3 (Eklundh & Jönsson, 2017; Jönsson & Eklundh, 2002, 2004). As suggested by Eklundh and Jönsson (2017), we duplicated the first and last year of data (i.e., 1982 and 2015) to calculate the metrics for the full time series (i.e., 36 years with 24 points per year for 864 data points). We were not concerned about spike removal because of the preprocessing by GIMMS; therefore, we used the Savitzky–Golay filter with a window size of 2 and no spike removal. Additional settings within TIMESAT included the following: season start and stop at 25% of the seasonal amplitude; 3 envelope iterations; and an adaption strength of 2. We accepted the default values for all other parameters. We calculated amplitude and integrated NDVI for each hunt area in each year from 1982 to 2016. We then calculated mean values of each of those two metrics at three temporal scales for each individual sheep: year of gestation, the first 3 years of life, and the entire lifetime of an individual.

### 2.2 Identifying changes in horn size through time

To evaluate the relative weight of support for our hypotheses, we assessed temporal trends in mean horn size, mean age, age‐specific horn size, and age‐specific horn size after accounting for environmental effects of harvested sheep within hunt areas. To assess age‐specific changes through time, we aggregated hunt areas and binned years where necessary to reach sufficient sample sizes (Monteith et al., 2013). We required a minimum sample size of 40 harvested animals within a given cohort for each hunt area. To reach minimum sample sizes for a hunt area, we first aggregated hunt areas based on geographic locations within states and provinces until we reached the minimum sample size of harvested animals within each year. The aggregation of hunt areas resulted in a sample size of 72 hunt areas. Next, we combined years where necessary to produce the temporal bins (hereafter referred to simply as year) that contained a minimum range of ages (minimum age ≤ 6 and maximum age ≥ 9) of harvested animals to enhance the accuracy of our estimation of the horn growth curve. To produce temporal bins with the minimum range of ages, we began with the earliest year and added samples from each subsequent year until the minimum range of ages was achieved (Monteith et al., 2013).

To identify temporal changes in mean horn size of harvested sheep within hunt areas, we used weighted linear regression with the mean year of data contained in each temporal bin as the predictor variable and sample size as the weighting factor. We assessed statistical significance of changes in mean horn size through time in each hunt area based on whether the 95% CIs for the year effect overlapped zero (du Prel, Hommel, Röhrig, & Blettner, 2009). To identify changes in the mean age of harvested sheep in each hunt area, we used the same model structure, but with mean age at harvest as the response variable.

We modeled horn growth curves of cohorts born between 1981 and 2004, and assessed age‐specific changes in horn size through time while accounting for environmental conditions at three temporal scales corresponding to the year a cohort of sheep was born. To ensure a sufficient range of ages to increase the accuracy of modeled horn growth curves, we did not consider cohorts born after 2004 in this analysis. To test for age‐specific changes in horn size, we used mixed‐effects models to estimate horn growth curves for cohorts born in each hunt area between 1981 and 2004 using the lme4 package in program R (Bates, Maechler, Bolker, & Walker, 2015). Rate and size of horn growth are dependent on age; thus, we included both age and the natural log of age as fixed effects to account for the nonlinear, but generally asymptotic, relationship between horn size and age (Monteith et al., 2018). Because each of these covariates was necessary for describing horn growth curves for birth cohorts of harvested sheep, we did not perform formal model selection. We also included fixed effects for subspecies and measurement type (e.g., “Rocky Mountain bighorn sheep, full score”), to account for differences in size of horns between subspecies and measurement types. Finally, we included a random intercept and uncorrelated random slopes for age and the natural log of age, grouped by hunt area nested within temporal bin (Zuur, Ieno, Walker, Saveliev, & Smith, 2009). This approach yielded a conditional estimate of the horn growth curve for animals in each temporal bin in each hunt area.

For each hunt area, we extracted the predicted size of 7‐year‐old males in each temporal bin from 1981 to 2004 using the modeled horn growth curves. We used predicted size at age 7 because mean age at harvest from all records was 7.3 (±2.1) years and the horn growth curve began to asymptote between ages 6 and 8 for most hunt areas. Our modeling approach allowed us to use data points from every age class to estimate horn growth curves, thus contributing to the predicted horn size of 7‐year‐old males in each cohort.

To assess age‐specific changes in horn size through time, we fit a simple linear regression for each hunt area. We used predicted horn size at age 7 from our first mixed‐effects model as the response variable, and the mean year of data in each temporal bin, weighted by the sample size within each temporal bin, as the predictor variable. Using predicted horn size at age 7 instead of mean horn size at age 7 allowed us to take advantage of the full dataset, using all data points to inform horn size at age 7 for each cohort, and overcame challenges associated with depending upon a sufficient number of 7‐year‐olds in any 1 year (Gillies et al., 2006; Long et al., 2016). We set the minimum sample size to 9 temporal bins for inclusion in the analysis of temporal trends for each hunt area. We assessed statistical significance of age‐specific changes in horn size based on whether the 95% CIs for the year effect in each hunt area overlapped zero (du Prel et al., 2009).

To test for environmental effects on horn size, we included environmental variables during different stages of life in the simple linear regression for each hunt area. For each individual hunt area, we modeled age‐specific changes in horn size with predicted horn size at age 7 as the response variable, and temporal bin and 6 environmental covariates as the predictor variables, weighted by the sample size in each temporal bin. For each hunt area, we evaluated all possible combinations of predictor variables (with year required in each model) and used AIC*_c_* to determine the best model for explaining changes in horn size through time (Doherty, White, & Burnham, 2012). We also required a minimum of 6 degrees of freedom for each model. For Rocky Mountain bighorn sheep, we included cumulative snow water equivalent during gestation, mean minimum temperature during gestation, mean NDVI amplitude during early life, mean winter precipitation during early life, mean integrated NDVI during life, and mean NDVI amplitude during life as environmental covariates. For desert bighorn sheep, we included cumulative snow water equivalent during gestation, mean minimum temperature during gestation, mean snow water equivalent during early life, mean winter precipitations during early life, mean winter precipitation during life, and mean minimum temperature in winter during life as environmental covariates. For temporal bins that included multiple cohorts, we weighted environmental covariates by the sample size of individuals within each year in a given temporal bin. After accounting for environmental effects, we assessed statistical significance of age‐specific changes in horn size based on whether the 95% CIs for the year effect in each hunt area overlapped zero (du Prel et al., 2009).

We developed a metric of potential strength of harvest‐based selection against fast‐growing horns to assess whether harvest pressure was sufficient to produce a measurable effect on the mean age at which a cohort was harvested. Ideally, to assess true selective pressure caused by harvest we would need to assess the number of males eligible for harvest in a population in relation to how many males were actually harvested, in addition to known measurements of horn size of all males in a population. Those data, however, were unavailable, so we developed a metric of selectivity based on the premise that under selective and intensive harvest, cohorts that produced larger, faster‐growing males would be harvested at younger ages relative to cohorts that produced smaller males. We regressed the mean age at which animals in a temporal bin were harvested against the predicted size of 7‐year‐olds in that temporal bin, weighted by the number of animals that were harvested in each temporal bin.

Finally, we categorized potential selective pressure imposed by harvest practices in each hunt area as weak, moderate, or strong based on morphometric size requirements for harvest and quotas for the majority of hunts that occurred between 1981 and 2016 (Mysterud, 2011). Harvest of mountain sheep primarily has been regulated in one or both of two ways across North America—morphometric size requirements or quotas. Harvest regulations across hunt areas were established by either a minimum horn size to be harvested or by a quota on the number of animals that could be harvested, or a combination of both a quota and some minimum size requirement for harvest. We characterized hunt areas with no morphometric size requirements and limited quotas as imposing weak selective pressure, hunt areas that had a morphometric size requirement and a limited quota as imposing moderate selective pressure, and hunt areas that had a morphometric size requirement and unlimited quotas as imposing strong selective pressure.

### 2.3 Simulation‐based assessment

To evaluate whether the modeling approach we developed would be capable of detecting changing patterns of horn growth given bias in harvest data (Pelletier, Festa‐Bianchet, & Jorgenson, 2012), we simulated 180 populations of mountain sheep that were subjected to varying degrees of harvest intensity (1%, 5%, 10%, and 20% harvest of males) and selectivity (low, medium, and high selection for horn size). We assessed changes in horn growth of harvested animals from populations that had increasing (*n* = 60), decreasing (*n* = 60), and stable (*n* = 60) horn size over time using an identical framework to our analyses of harvest records. A detailed description of the simulation analysis is provided in Appendix S3.

## 3 RESULTS

We evaluated 24,786 records of mountain sheep harvested in 72 hunt areas in nine states in the United States and one Canadian province between 1981 and 2016. Mean horn size of harvested male sheep changed during the study period in 38.9% (*n* = 28) of hunt areas, with declines evident in 26.4% (*n* = 19) of hunt areas, and 12.5% (*n* = 9) exhibiting increases in horn size through time (Table 1). Mean age of harvested males changed in 19.4% (*n* = 14) of hunt areas, with age declining in 8.3% (*n* = 6) and increasing in 11.1% (*n* = 8) of hunt areas through time (Table 2).

**Table 1:** Mean and range of change (cm/year) in predicted horn size (cm) of 7‐year‐old male mountain sheep (Rocky Mountain bighorn sheep and desert bighorn sheep) as a function of horn size metric (full score or length + base score) in cohorts born between 1981 and 2004 in 72 hunt areas across western United States and Canada

Subspecies	Trend (horn size metric)	Mean (range)
Rocky Mountain bighorn sheep	Full score	−0.02 (−0.24 to 0.37)
	Length + base score	−0.04 (−0.26 to 0.21)
Desert bighorn sheep	Full score	0.015 (−0.52 to 0.30)
	Length + base score	−0.135 (−0.28 to 0.01)

**Table 2:** Number of hunt areas that exhibited decreases, increases, or no change in mean age at harvest and mean horn size from 1981 to 2016, and hunt areas that exhibited decreases, increases, or no change in predicted horn size of 7‐year‐old males before accounting for the environment, and predicted horn size of 7‐year‐old males after accounting for the environment from cohorts born from 1981 to 2004 in hunt areas of mountain sheep across western United States and Canada

Trends	Decreasing	Increasing	Stable	Total
Mean age	6	9	57	72
Mean horn size	19	8	45	72
Predicted horn size of 7‐year‐old males	18	6	48	72
Predicted horn size of 7‐year‐old males with environment	16	6	50	72

**Figure 3:**
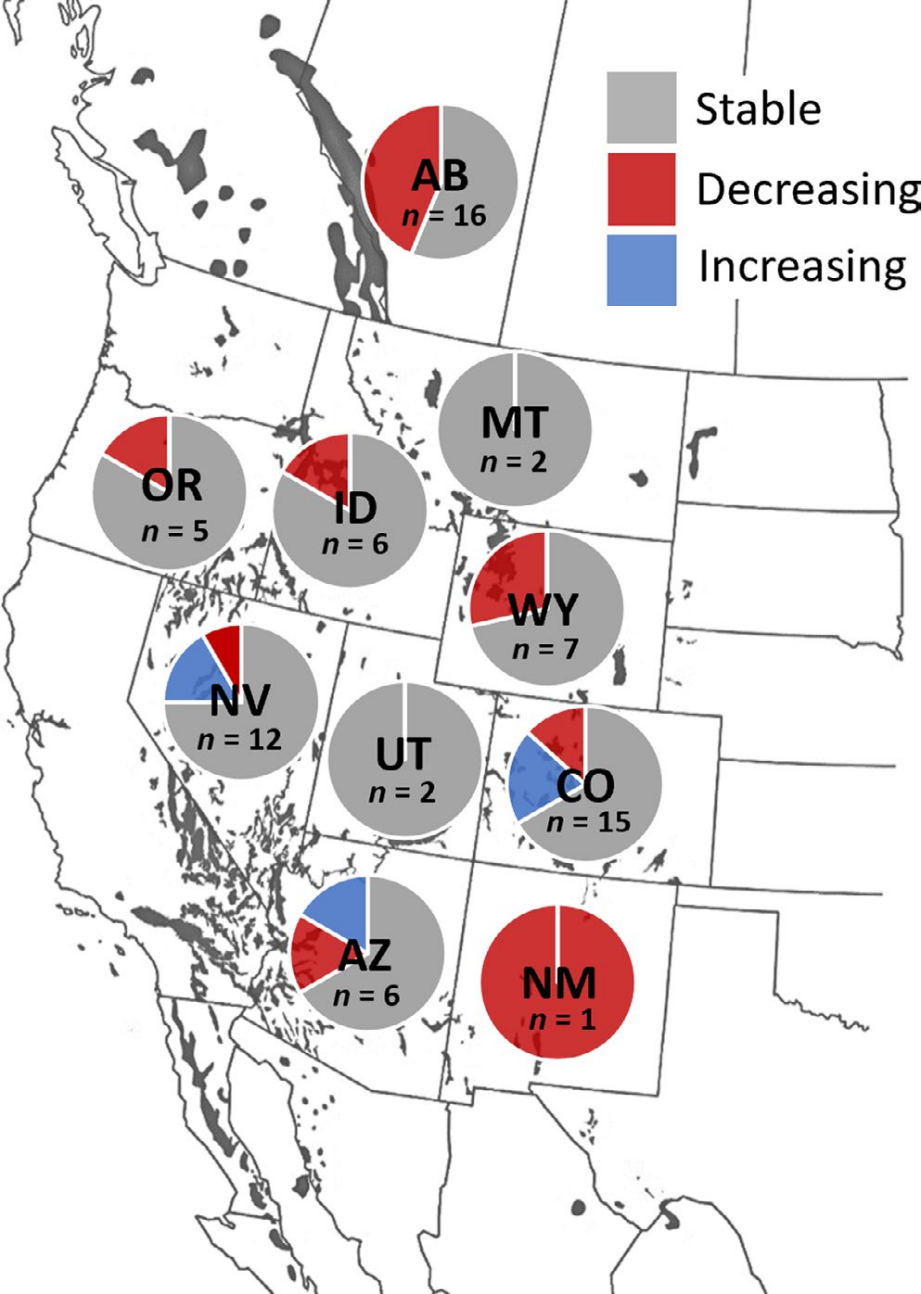
Proportion of hunt areas in each U.S. state or Canadian province that has either stable, increasing, or decreasing horn size after accounting for both age and environmental conditions in cohorts born from 1981 to 2004. Areas with no temporal change are represented with gray, areas with decreases in horn size are represented with red, and areas with increases in horn size are represented with blue. Current bighorn sheep range is represented in black. Sample size for each state or province represents the number of hunt areas. State and provincial codes: AB—Alberta, AZ—Arizona, CO—Colorado, ID—Idaho, MT—Montana, NM—New Mexico, NV—Nevada, OR—Oregon, UT—Utah, WY—Wyoming

**Figure 4:**
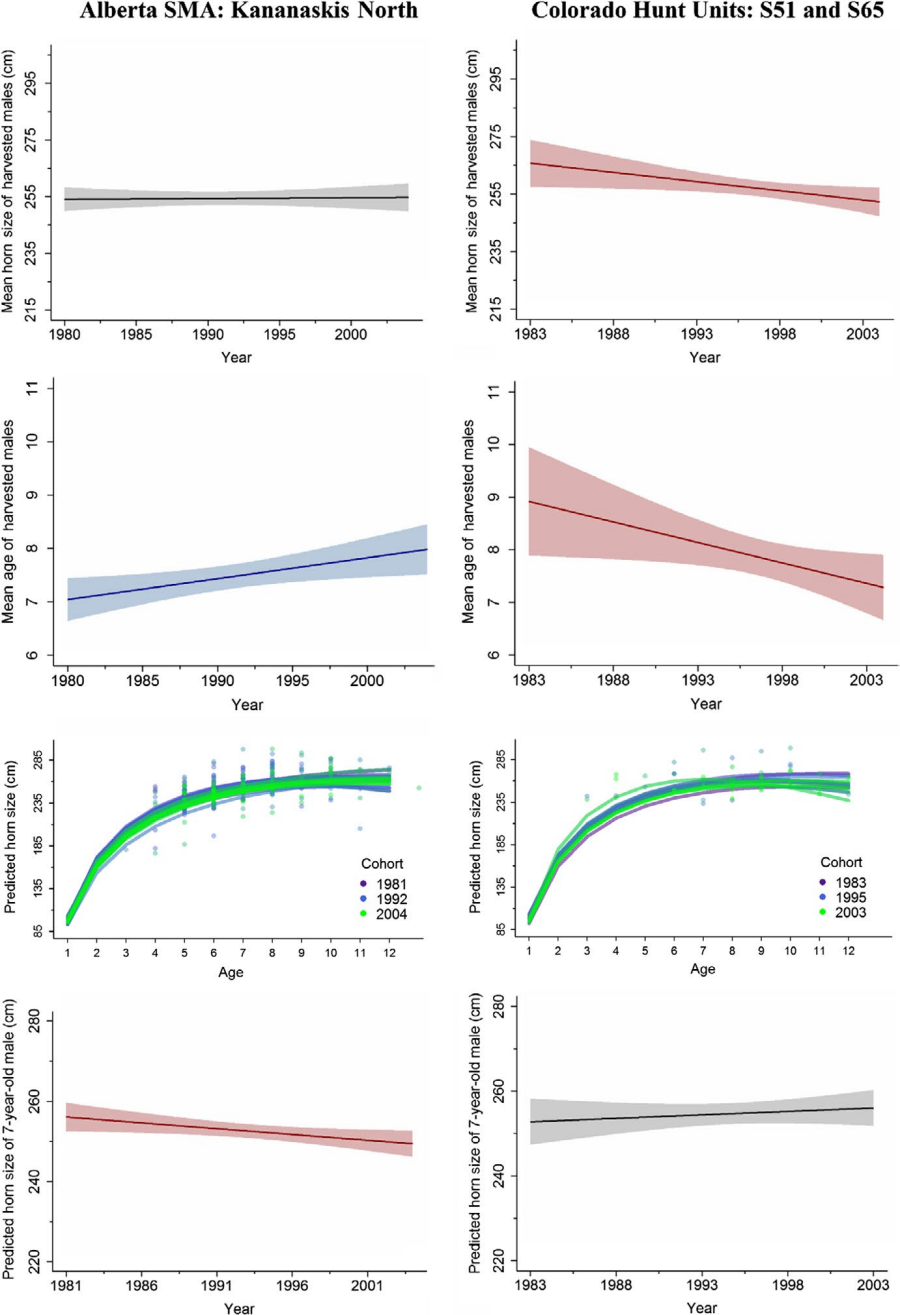
Trend lines and confidence intervals for change in mean age (years) of cohorts of harvested males from 1981 to 2004, change in mean horn size (cm; based on length‐base score) of cohorts of harvested males from 1981 to 2004, horn growth curves (cm) of cohorts born between 1981 and 2004, and trends in predicted horn size (cm) of 7‐year‐old males from 1981 to 2004 in 2 example hunt areas: one with no change in horn size (Colorado hunt units S51 and S65) and one with declining horn size (Alberta Sheep Management Area—Kananaskis North) of 7‐year‐old males. Significant negative trends are represented by red confidence intervals, significant positive trends are represented by blue confidence intervals, and no temporal change is represented by gray confidence intervals

Based on predicted, cohort‐specific curves of horn growth in each hunt area, horn size of 7‐year‐old males born between 1981 and 2004 did not change in 66.7% (*n* = 48) of hunt areas, declined in 25.0% (*n* = 18), and increased in 8.3% (*n* = 6) of hunt areas (Table 2). Environmental effects explained changes in the predicted horn size of 7‐year‐old males in 22.2% (*n* = 4) of hunt areas in which horn growth declined. Furthermore, after accounting for the effects of environmental variation, 8.3% (*n* = 2) of hunt areas that previously exhibited no temporal changes in horn size showed decreases in the predicted horn size of 7‐year‐old males. After accounting for age and environmental effects, predicted horn size of 7‐year‐old males did not change in 69.4% (*n* = 50) of hunt areas, decreased in 22.2% (*n* = 16; $\overset{¯}{x}$ = −0.19 cm/year [−0.09 to 0.52]), and increased in 8.3% (*n* = 6; $\overset{¯}{x}$ = 0.23 cm/year [0.15–0.37]) of hunt areas (Figures 3 and 4).

In 5.6% (*n* = 4) of hunt areas, males from cohorts with faster‐growing horns were harvested at a younger age than males from slower‐growing cohorts, and there was a concomitant decrease in the predicted horn size of 7‐year‐old males through time in 50% of those hunt areas. For the 16 hunt areas in which harvest had the strongest potential to impose selective pressure based on characteristics of the harvest regime, 43.75% (*n* = 7) exhibited declines in the predicted size of 7‐year‐old males through time. In the 22 hunt areas with moderate potential for harvest to impose selective pressure, 13.6% (*n* = 3) exhibited declines in the predicted size of 7‐year‐old males through time. In the 34 hunt areas that had the weakest potential for harvest to impose selective pressure, 18% (*n* = 6) exhibited declines in the predicted size of 7‐year‐old males through time.

We evaluated harvest data from 180 simulated populations of bighorn sheep. For hunt areas that had simulated increases (*n* = 60) or decreases (*n* = 60) in horn size over time, regardless of harvest intensity or selectivity, we detected corresponding increases or decreases in horn size of 7‐year‐old males in 100% of hunt areas. In 15% of hunt areas with simulated stability in horn size over time (*n* = 5), we detected a significant relationship (both positive and negative) between horn size of 7‐year‐old males and year (Appendix S3).

## 4 DISCUSSION

Research and media attention associated with the effects of harvest on wildlife over the past few decades have yielded increased interest among scientists, wildlife managers, and the public in understanding the consequences of harvest (Festa‐Bianchet, 2017; Festa‐Bianchet & Mysterud, 2018; Heffelfinger, 2018). Nevertheless, most research in terrestrial systems demonstrating potential evolutionary consequences of harvest has been limited to a management unit comprised of a single population of mountain sheep (Coltman et al., 2003; Pigeon et al., 2016), which makes extrapolating results from those studies to larger geographic and temporal scales difficult (but see Festa‐Bianchet, Pelletier, Jorgenson, Feder, & Hubbs, 2014). We analyzed harvest records that included horn size and age data for mountain sheep collected over 35 years and spanning much of the range of mountain sheep in North America to elucidate the relative influence of demography, harvest, and the environment on horn size and growth through time. In nearly 70% of hunt areas, horn size of harvested males remained stable during the study period. Where changes in horn size occurred, they typically were explained most parsimoniously by changes in demography, lending support to the Demographic Shift Hypothesis. For horn growth, changes were related to environmental factors (e.g., climate and forage availability) in some instances, lending some support to the Environmental Effects Hypothesis. After accounting for age and environmental effects, age‐specific horn size of mountain sheep was either stable or increasing in the majority (~78%) of hunt areas in the United States and Canada. The remaining hunt areas (~22%) exhibited declines in age‐specific horn size through time, less than half of which were associated with metrics of harvest intensity and selectivity consistent with the Selective Harvest Hypothesis and associated potential for evolutionary change.

Changes in age structure can have important effects on growth rates of populations (Festa‐Bianchet et al., 2014; Schindler, Festa‐Bianchet, Hogg, & Pelletier, 2017) and can underpin changes in size of harvested individuals (Monteith et al., 2013). Horn size in mountain sheep is dependent on age (Bergeron, Festa‐Bianchet, Hardenberg, & Bassano, 2008; Bunnell, 1978; Monteith et al., 2018), but because the relationship between horn size and age is nonlinear and begins to asymptote between 6 and 8 years of age (Monteith et al., 2018), there may not be a linear relationship between changes in the horn size of harvested animals and their age. Indeed, we detected a higher percentage of changes in horn size of harvested sheep (38.9%) compared with changes in age of harvested sheep (19%), yet over half of the areas that did exhibit changes in horn size did not show corresponding changes in age‐specific horn size over time.

Growth of secondary sexual characteristics that are nonessential to survival is influenced strongly by the availability of resources, not only to the growing male (Monteith et al., 2018), but also to the mother during gestation and lactation (Büntgen et al., 2014; Jorgenson, Festa‐Bianchet, & Wishart, 1998; Michel et al., 2016; Monteith et al., 2009; Toïgo, Gaillard, & Michallet, 1999). In accordance with the Environmental Effects Hypothesis, indices of climate and forage availability explained declines in predicted horn size of 7‐year‐old males in roughly 22.2% (4 of 18 hunt areas) of hunt areas, while revealing changes in 3.7% (2 of 54 hunt areas) of hunt areas that otherwise did not exhibit temporal change in age‐specific horn size (Table S1). Environmental conditions and their effects on resource availability can mask or accentuate underlying temporal trends in horn growth. Environmental conditions often are evident in annual growth of horns (Giacometti, Willing, & Defila, 2002), which may provide an index to when, and to which environmental conditions, individuals were exposed to during their lives. Moreover, variation in annual growth of horns has been positively linked to warm spring temperatures, early snow melt, and early plant green‐up (Büntgen et al., 2014). Thus, environmental conditions can have important implications for both size and growth of horns throughout an individual's life.

Although we attempted to account for the influence of climate and forage availability by including broad‐scale indices in our models of horn size, several other factors also can influence nutrition (and thus horn growth) that we were unable to account for because of the scale of our analyses and the availability of relevant data, among which are animal density, disease, and translocations. Population density has direct implications for nutrition (Bowyer et al., 2014; Monteith et al., 2018) and can have stronger effects on horn size than underlying genetic change (Festa‐Bianchet, 2017; Jorgenson et al., 1998; Kruuk et al., 2002; Pigeon et al., 2016). High densities can result in increased competition for resources, decreased nutritional condition, and a subsequent decrease in horn growth (Festa‐Bianchet, 2017; Jorgenson et al., 1998; Monteith et al., 2018). Unfortunately, reliable estimates of density or nutritional condition do not exist in most hunt areas we analyzed. In addition, the interaction between nutrition and disease may have important implications for population density (Monteith et al., 2018). Mountain sheep have a long history of epizootic respiratory disease throughout North America, beginning as early as the turn of the 20th century (Grinnell, 1928). Such outbreaks can result in marked population declines and thus large reductions in density (Cassirer et al., 2018; Monello, Murray, & Cassirer, 2001; Shannon et al., 2014). Following an outbreak of pneumonia, when populations persist but remain chronic carriers of pathogens associated with pneumonia (Cassirer et al., 2018), the degree to which infection may interact with nutrition to affect not only resilience but also allocation to traits such as horn growth remains unclear (Downs & Stewart, 2014; Downs, Stewart, & Dick, 2015; Monteith et al., 2018). Unfortunately, consistent data on mortality from disease outbreaks were not available for most hunt areas, and we could not account for potential effects of disease and population density in our analyses.

Translocation and reintroduction efforts have been an important tool for the recovery and management of mountain sheep throughout North America (Bleich, Sargeant, & Wiedmann, 2018; Hurley, Brewer, & Thornton, 2015; Krausman, 2000; Singer, Papouchis, & Symonds, 2000). Introduction of new animals into a population through translocations can influence demography and density, and has potential to introduce new genes, disease, and individuals that differ in nutritional condition. Translocation of new individuals into an area has the potential to provide a buffer against harvest‐induced evolution of horn size through the introduction of new genetic material. Prior translocation of novel genetic stock is an important confounding factor when attempting to parse the effects of harvest on horn size; yet, translocated individuals are most often sourced from populations already exposed to some level of harvest pressure and hunter selection. Movement of animals from one harvested population to another harvested population, therefore, likely would not introduce “genetically superior” individuals, or buffer populations from the effects of harvest (Pelletier, Festa‐Bianchet, Jorgenson, Feder, & Hubbs, 2014), in part because genetic contributions to horn size may be overridden by nutrition (Monteith et al., 2018). A translocated female that differs markedly in condition from the average female in the translocated population may produce a son that reflects conditions where she was moved from; as a result, her son may have either larger or smaller horns than the average male born into the translocated population (Michel et al., 2016; Monteith et al., 2009). Nutritional condition, however, is a product of the environment in which an individual resides, and translocated individuals would be expected to adjust to environmental conditions in their new area quickly, and it is unlikely that condition of a translocated female would differ from the rest of the population for more than a single breeding season (Monteith et al., 2014; Parker, Barboza, & Gillingham, 2009). Alternatively, when translocated males are available for harvest, their horns may reflect the environment in which they developed (i.e., their natal range), thus adding “noise” to the relationship between forage conditions and horn size in populations containing translocated individuals.

Although our ability to address certain mechanisms explicitly was hampered by the scale of our analyses, addressing questions of selective harvest at such a broad scale yielded a robust sample wherein biologically meaningful changes are detectable (Monteith et al., 2013). We acknowledge that in some instances, more detailed data could have helped account for changes through time; however, we hope our results provide a foundation on which to build subsequent inquiry on the evolutionary effects of harvest. Moreover, our results may yield an assessment of trajectories in horn size and links to harvest at a scale that has not been accomplished yet. Further, results of our simulation analyses indicate that the modeling approach we used provided a valid assessment for detecting changes in growth of horns in populations over time. Although we did detect anomalous changes in 15% of simulated populations that had stable horn growth over time, in the vast majority (85%) of simulated hunt areas with stable horn size there was no detectable change over time and we detected change in all hunt areas where change in horn growth occurred. Notably, based on our simulations, highly conservative or selective harvest did not preclude our ability to detect meaningful changes in horn growth when they were present (Appendix S3).

Mountain sheep are one of the most coveted big game species in the world (Monteith et al., 2018). Most hunters wait decades for the chance to harvest a bighorn sheep, and many state agencies will permit hunters to harvest only one male sheep during that hunter's lifetime. The conservative harvest practices that characterize management of mountain sheep throughout most of North America likely produce strong selection for weapon size (i.e., large horns) by hunters compared with other species of big game in North America. Declines in predicted horn size of 7‐year‐old males after accounting for age and remotely sensed metrics of climate and forage availability were evident in 22% (*n* = 16) of hunt areas in our study. Although past studies have focused primarily on horn length (Festa‐Bianchet et al., 2014) as opposed to a metric of horn size, consistent with past research, hunt areas with simultaneously selective and intensive harvest regimes (i.e., stronger potential for selective pressure) were more likely to exhibit age‐specific declines in horn size than were hunt areas with less selective and less intensive harvest regimes (Festa‐Bianchet et al., 2014; Pigeon et al., 2016). We detected declines in horn growth in less than half of hunt areas where harvest was regulated solely by a morphological criterion (i.e., horn length), which supports the notion that an evolutionary effect is more likely to occur in areas with simultaneously high selectivity and harvest intensity, lending some support for the selective harvest hypothesis (Figure 3). Nevertheless, our results also indicate that changes in horn growth are not implicit even in the face of highly selective and intensive harvest, likely because of the myriad other factors that influence the manifestation of evolutionary effects (Heffelfinger, 2018).

Harvest‐induced evolution is often cited as the underlying force behind changes in phenotypic characteristics of populations (Allendorf & Hard, 2009; Conover, Munch, & Arnott, 2009; Darimont et al., 2009), and in extreme instances has been suggested to result in extinction of species (Knell & Martínez‐Ruiz, 2017). In the United States and Canada, hunting remains a fundamental part of wildlife conservation and management (Geist, Mahoney, & Organ, 2001; Heffelfinger, Geist, & Wishart, 2013; Leopold, 1987), but the sustainability of harvest practices and public perceptions of harvest likely will dictate the viability of hunting as a management tool in the future (Allendorf et al., 2008; Heffelfinger, 2018; Kuparinen & Festa‐Bianchet, 2017). Thus, disentangling biological consequences from perceived consequences of harvest is imperative to successful management and conservation of wildlife, as is the effective communication of research on the consequences of harvest to managers, biologists, and the public (Crosmary, Côté, &, Fritz, 2015; Hurley et al., 2015; Simon, 2016). Unlike what has been promoted in the popular literature, most harvest practices for mountain sheep that are implemented by state and provincial agencies have not resulted in negative changes in horn growth patterns over time, as evidenced by stable or increasing trends in horn growth over nearly 3 decades in the majority of hunt areas throughout the western United States and Canada. In areas where declines in horn growth have occurred and are consistent with the potential for evolutionary changes from highly selective and liberal harvest, certain management strategies could reduce the potential for selective pressure to produce undesirable changes in horn growth over time. In mountain sheep, management strategies that limit harvest to animals that have obtained a minimum horn size in conjunction with liberal harvest quotas in situations where animals are vulnerable to harvest (i.e., high harvest intensity) may be less likely to maintain horn size and growth over time. Alternatively, management that limits harvest by a quota instead of a morphological criterion appears to result in stability of horn size. In areas where evidence suggests that harvest could be contributing to declines in horn growth, changes to management regulations may help to buffer or slow the potential for evolutionary changes (Pigeon et al., 2016). For example, removal of size requirements for harvest (Mysterud, 2011), reducing harvest pressure (Mysterud, 2011), or we propose, defining a legal male based on age instead of a morphological criterion all would reduce selective pressure operating on heritable traits. Further investigation of hunt areas in which we detected declines or increases in horn growth after accounting for age and the environment likely will elucidate additional factors that result in population‐level changes in horn growth. Although highly intensive and selective harvest can result in phenotypic changes with evolutionary underpinnings (Pigeon et al., 2016), harvest does not inherently produce phenotypic changes in populations, and under conservative harvest practices, selective harvest may occur without deleterious effects on horn growth through time.

## CONFLICT OF INTEREST

None declared.

## Data Availability

The data that support the findings of this study were provided by state and provincial agencies. Authors do not have rights to distribute or share these data. Request for data can be made to individual agencies.
