# Supplementary material for: Hunting and mountain sheep: Do current harvest practices affect horn growth?
Source: Evol Appl. 2019 Jul 29;12(9):1823–36. doi: 10.1111/eva.12841 (PMC6752155; doi:10.1111/eva.12841)
Supplement: Supplementary file 1 [file EVA-12-1823-s001.docx]

**APPENDIX**

**Appendix S1. Changes in mean age and mean horn size, predicted horn size of 7-year-old males before and after accounting for the environment, and metrics of selectivity of each hunt area.**

Table S1. Model output for each analysis by individual hunt area from 1981 to 2015 in 10 states and provinces in the U.S.A and Canada. Hunt regulations indicate the level of potential harvest intensity and severity, categorized as weak, moderate, or strong based on morphometric size requirements and quotas. Estimates (β) and confidence intervals for horn size trends represents the change, and upper and lower confidence limits of mean horn size in hunt areas through time. Estimate and confidence intervals for age trends represents the change and upper and lower confidence limits of mean age in hunt areas through time. Asterisks (*) on the beta coefficient indicate significance based on the 95% confidence interval for each analysis.

| **State/Province** | **Hunt Area** | **Harvest**  **Intensity** | **n years** | **n records** |  | **Trends in Horn Size** | | | |  | **Trends in Age** | | | |
| --- | --- | --- | --- | --- | --- | --- | --- | --- | --- | --- | --- | --- | --- | --- |
|  |  |  |  |  |  |  | | **Confidence Interval** | |  |  | | **Confidence Interval** | |
|  |  |  |  |  |  | **Estimate** | **R^2^** | **Lower** | **Upper** |  | **Estimate** | **R^2^** | **Lower** | **Upper** |
| Alberta | AB-BowValley-Ghost-North | Strong | 21 | 227 |  | -0.15* | 0.18 | -0.30 | <0.01 |  | -0.02 | 0.05 | -0.06 | 0.02 |
|  | AB-BowValley-Ghost-South | Strong | 12 | 203 |  | -0.33* | 0.48 | -0.48 | -0.17 |  | -0.01 | 0.02 | -0.04 | 0.02 |
|  | AB-Cadomin | Strong | 24 | 808 |  | 0.07 | 0.06 | -0.05 | 0.19 |  | 0.02 | 0.05 | -0.02 | 0.05 |
|  | AB-Clearwater-Ram | Strong | 16 | 199 |  | <0.01 | <0.01 | -0.18 | 0.17 |  | <0.01 | <0.01 | -0.05 | 0.04 |
|  | AB-Clearwater-Ram-420 | Strong | 24 | 368 |  | -0.11* | 0.18 | -0.21 | -0.01 |  | <0.01 | <0.01 | -0.03 | 0.03 |
|  | AB-Clearwater-Ram-422 | Strong | 21 | 333 |  | -0.03 | 0.02 | -0.11 | 0.06 |  | 0.01 | 0.03 | -0.02 | 0.05 |
|  | AB-Clearwater-Ram-South | Strong | 22 | 325 |  | -0.16* | 0.17 | -0.31 | -0.01 |  | -0.01 | 0.01 | -0.04 | 0.03 |
|  | AB-Kananaskis-North | Strong | 24 | 344 |  | 0.01 | <0.01 | -0.12 | 0.14 |  | 0.04* | 0.23 | 0.01 | 0.07 |
|  | AB-Kananaskis-South | Strong | 22 | 437 |  | -0.06 | 0.07 | -0.15 | 0.04 |  | 0.03 | 0.13 | <0.01 | 0.05 |
|  | AB-Livingstone-North | Strong | 11 | 176 |  | 0.11 | 0.14 | -0.01 | 0.24 |  | 0.04* | 0.34 | 0.01 | 0.06 |
|  | AB-Nordegg-Chungo-North | Strong | 24 | 468 |  | -0.11* | 0.28 | -0.19 | -0.03 |  | <0.01 | 0.01 | -0.02 | 0.03 |
|  | AB-Nordegg-Chungo-South | Strong | 13 | 187 |  | -0.04 | 0.02 | -0.16 | 0.08 |  | -0.01 | 0.03 | -0.04 | 0.02 |
|  | AB-Torrens | Strong | 20 | 428 |  | -0.01 | <0.01 | -0.12 | 0.11 |  | -0.03 | 0.05 | -0.08 | 0.02 |
|  | AB-Westcastle-Yarrow | Strong | 12 | 219 |  | 0.43* | 0.52 | 0.25 | 0.61 |  | 0.11* | 0.55 | 0.07 | 0.16 |
|  | AB-Willmore-North | Strong | 24 | 381 |  | -0.08* | 0.18 | -0.16 | -0.01 |  | 0.01 | 0.03 | -0.01 | 0.03 |
|  | AB-Willmore-South | Strong | 23 | 472 |  | -0.11* | 0.25 | -0.19 | -0.03 |  | <0.01 | <0.01 | -0.02 | 0.02 |
| Arizona | AZ-12.13 | Weak | 12 | 158 |  | -0.23* | 0.21 | -0.43 | -0.04 |  | -0.03 | 0.06 | -0.09 | 0.02 |
|  | AZ-15.16 | Weak | 21 | 683 |  | -0.18* | 0.27 | -0.31 | -0.05 |  | <0.01 | <0.01 | -0.02 | 0.02 |
|  | AZ-37.39.33.32.31 | Weak | 13 | 155 |  | -0.04 | 0.01 | -0.24 | 0.17 |  | <0.01 | <0.01 | -0.03 | 0.03 |
|  | AZ-40.46 | Weak | 18 | 236 |  | -0.11 | 0.07 | -0.30 | 0.07 |  | -0.02 | 0.03 | -0.06 | 0.02 |
|  | AZ-44 | Weak | 18 | 467 |  | 0.27* | 0.37 | 0.12 | 0.43 |  | <0.01 | <0.01 | -0.03 | 0.03 |
|  | AZ-6.22.24 | Weak | 12 | 75 |  | 0.18 | 0.15 | -0.02 | 0.38 |  | 0.02 | 0.02 | -0.04 | 0.09 |
| Colorado | CO-S01.18.19.37.40.57.58.73 | Moderate | 12 | 155 |  | <0.01 | <0.01 | -0.31 | 0.30 |  | 0.02 | 0.01 | -0.06 | 0.10 |
|  | CO-S02.59.67.74.77 | Moderate | 12 | 76 |  | 0.01 | <0.01 | -0.33 | 0.36 |  | -0.01 | <0.01 | -0.10 | 0.08 |
|  | CO-S03.04.23.27.32.41 | Moderate | 22 | 587 |  | -0.12 | 0.08 | -0.29 | 0.05 |  | 0.04 | 0.12 | -0.01 | 0.08 |
|  | CO-S05.06.34.46.60.72 | Moderate | 20 | 269 |  | -0.25* | 0.26 | -0.44 | -0.07 |  | <0.01 | <0.01 | -0.05 | 0.06 |
|  | CO-S07.49.68 | Moderate | 9 | 112 |  | 0.32 | 0.07 | -0.21 | 0.84 |  | 0.06 | 0.14 | -0.01 | 0.13 |
|  | CO-S08.09.35.50 | Moderate | 21 | 312 |  | -0.21* | 0.20 | -0.39 | -0.03 |  | -0.03 | 0.10 | -0.06 | 0.01 |
|  | CO-S10.20.55 | Moderate | 12 | 137 |  | -0.13 | 0.02 | -0.54 | 0.28 |  | 0.04 | 0.03 | -0.06 | 0.15 |
|  | CO-S11.12.17.39.47.66 | Moderate | 21 | 430 |  | 0.08 | 0.05 | -0.07 | 0.23 |  | 0.05* | 0.21 | 0.01 | 0.09 |
|  | CO-S13.25.44 | Moderate | 16 | 152 |  | 0.23 | 0.13 | -0.03 | 0.50 |  | 0.02 | 0.01 | -0.05 | 0.08 |
|  | CO-S15.16.28.71 | Moderate | 15 | 160 |  | 0.23 | 0.14 | -0.01 | 0.48 |  | 0.01 | <0.01 | -0.05 | 0.06 |
|  | CO-S21.22.33.36.52.53 | Moderate | 13 | 151 |  | 0.45* | 0.33 | 0.17 | 0.74 |  | 0.10* | 0.21 | 0.01 | 0.18 |
|  | CO-S26.54.69.70 | Moderate | 14 | 113 |  | 0.17 | 0.08 | -0.10 | 0.43 |  | 0.06 | 0.10 | -0.02 | 0.13 |
|  | CO-S29.30.31 | Moderate | 11 | 105 |  | 0.03 | <0.01 | -0.32 | 0.38 |  | 0.06 | 0.12 | -0.01 | 0.13 |
|  | CO-S38.48.61 | Moderate | 14 | 106 |  | 0.28 | 0.16 | -0.01 | 0.56 |  | 0.06 | 0.11 | -0.02 | 0.15 |
|  | CO-S51.65 | Moderate | 14 | 126 |  | -0.25* | 0.23 | -0.47 | -0.03 |  | -0.08* | 0.22 | -0.15 | -0.01 |
| Idaho | ID-17.21.21A.28 | Moderate | 10 | 114 |  | -0.20 | 0.14 | -0.41 | 0.02 |  | <0.01 | <0.01 | -0.04 | 0.04 |
|  | ID-19.20.20A.23.24.25 | Moderate | 15 | 233 |  | -0.17* | 0.23 | -0.30 | -0.04 |  | -0.06* | 0.30 | -0.10 | -0.02 |
|  | ID-26 | Moderate | 15 | 112 |  | -0.07 | 0.01 | -0.34 | 0.20 |  | -0.01 | 0.01 | -0.07 | 0.04 |
|  | ID-36.36A.36B.50 | Moderate | 15 | 172 |  | -0.06 | 0.03 | -0.22 | 0.10 |  | 0.02 | 0.02 | -0.03 | 0.07 |
|  | ID-37.37A.51 | Moderate | 10 | 63 |  | 0.13 | 0.07 | -0.08 | 0.34 |  | 0.04 | 0.10 | -0.01 | 0.10 |
|  | ID-40.41.42.46.47 | Moderate | 20 | 473 |  | <0.01 | <0.01 | -0.13 | 0.13 |  | 0.02 | 0.03 | -0.02 | 0.06 |
| Montana | MT-122.124.121.123.100.102.101 | Weak | 9 | 393 |  | -0.06 | 0.02 | -0.23 | 0.12 |  | <0.01 | <0.01 | -0.06 | 0.06 |
|  | MT-210.212.213.261.250.270.340 | Moderate | 13 | 421 |  | -0.05 | 0.02 | -0.24 | 0.13 |  | 0.04 | 0.04 | -0.04 | 0.12 |
| New Mexico | NM-North | Weak | 16 | 160 |  | -0.06 | 0.02 | -0.29 | 0.16 |  | 0.06 | 0.10 | -0.03 | 0.14 |
| Nevada | NV-12.14.32.33.34 | Weak | 19 | 360 |  | 0.55* | 0.45 | 0.27 | 0.83 |  | -0.02 | 0.06 | -0.06 | 0.02 |
|  | NV-161.162.163.173 | Weak | 16 | 208 |  | -0.01 | <0.01 | -0.28 | 0.27 |  | -0.03 | 0.08 | -0.07 | 0.01 |
|  | NV-202.204.205 | Weak | 11 | 103 |  | 0.49 | 0.11 | -0.19 | 1.18 |  | <0.01 | <0.01 | -0.07 | 0.08 |
|  | NV-206.207.208.211.213.212 | Weak | 17 | 314 |  | 0.57* | 0.40 | 0.27 | 0.88 |  | 0.07* | 0.39 | 0.03 | 0.10 |
|  | NV-244.268.286 | Weak | 22 | 475 |  | -0.21 | 0.14 | -0.44 | 0.01 |  | -0.03 | 0.16 | -0.05 | <0.01 |
|  | NV-252.253.254 | Weak | 12 | 180 |  | 0.34* | 0.20 | 0.03 | 0.65 |  | 0.07* | 0.39 | 0.03 | 0.12 |
|  | NV-261.262 | Weak | 17 | 172 |  | -0.06 | 0.01 | -0.31 | 0.19 |  | <0.01 | <0.01 | -0.03 | 0.03 |
|  | NV-263.264.265.266 | Weak | 15 | 320 |  | 0.25* | 0.18 | 0.02 | 0.49 |  | <0.01 | <0.01 | -0.03 | 0.04 |
|  | NV-271.267.272.243 | Weak | 21 | 406 |  | -0.40* | 0.28 | -0.68 | -0.13 |  | -0.02 | 0.11 | -0.05 | <0.01 |
|  | NV-280.281.282.283.284 | Weak | 19 | 267 |  | -0.15 | 0.05 | -0.43 | 0.14 |  | 0.06* | 0.31 | 0.02 | 0.10 |
|  | NV-31.35.51 | Weak | 18 | 219 |  | -0.34 | 0.14 | -0.71 | 0.04 |  | -0.05 | 0.14 | -0.12 | 0.01 |
|  | NV-44.45.153.181.182.183.184 | Weak | 13 | 299 |  | 0.28* | 0.23 | 0.04 | 0.52 |  | 0.04 | 0.11 | -0.01 | 0.08 |
| Oregon | OR-Deschutes | Weak | 9 | 80 |  | -0.16 | 0.03 | -0.68 | 0.36 |  | -0.15 | 0.26 | -0.29 | <0.01 |
|  | OR-GrandeRonde | Weak | 16 | 182 |  | -0.26 | 0.12 | -0.58 | 0.05 |  | 0.01 | 0.02 | -0.03 | 0.06 |
|  | OR-JohnDay | Weak | 13 | 201 |  | 0.09 | 0.01 | -0.33 | 0.51 |  | 0.02 | 0.02 | -0.04 | 0.08 |
|  | OR-Klamath | Weak | 24 | 552 |  | -0.26* | 0.35 | -0.41 | -0.11 |  | <0.01 | <0.01 | -0.04 | 0.03 |
|  | OR-Malheur | Weak | 23 | 521 |  | -0.17 | 0.11 | -0.39 | 0.04 |  | -0.02 | 0.05 | -0.06 | 0.02 |
| Utah | UT-12.13.14.15.26 | Weak | 13 | 383 |  | -0.22* | 0.29 | -0.41 | -0.03 |  | -0.24* | 0.76 | -0.31 | -0.17 |
|  | UT-8.10.11 | Weak | 12 | 219 |  | -0.25* | 0.25 | -0.49 | <0.01 |  | -0.17* | 0.69 | -0.23 | -0.10 |
| Wyoming | WY-ClarksForkTroutPeak | Weak | 24 | 720 |  | <0.01 | <0.01 | -0.13 | 0.12 |  | -0.04* | 0.26 | -0.08 | -0.01 |
|  | WY-FrancsPeak | Weak | 24 | 1286 |  | -0.07 | 0.11 | -0.16 | 0.02 |  | <0.01 | <0.01 | -0.02 | 0.02 |
|  | WY-Laramie | Weak | 16 | 120 |  | 0.43* | 0.21 | 0.05 | 0.82 |  | 0.05 | 0.07 | -0.03 | 0.12 |
|  | WY-TargheeDarbyJackson | Weak | 17 | 285 |  | -0.35* | 0.19 | -0.65 | -0.04 |  | -0.07 | 0.15 | -0.13 | <0.01 |
|  | WY-WaipitiRidge | Weak | 24 | 920 |  | -0.19* | 0.30 | -0.31 | -0.07 |  | -0.08* | 0.57 | -0.11 | -0.05 |
|  | WY-WhiskeyMtn | Weak | 23 | 737 |  | -0.18* | 0.38 | -0.28 | -0.08 |  | -0.03 | 0.14 | -0.06 | <0.01 |
|  | WY-YontsPeak | Weak | 22 | 717 |  | 0.01 | <0.01 | -0.11 | 0.13 |  | -0.02 | 0.12 | -0.05 | <0.01 |
|  |  |  |  |  |  |  |  |  |  |  |  |  |  |  |

Table S2. Model output for each analysis by individual hunt area from 1981 to 2015 in 10 states and provinces in the U.S.A and Canada. Harvest intensity indicates the level of potential harvest intensity and severity, categorized as weak, moderate, or strong based on morphometric size requirements and quotas. Estimate and confidence intervals for predicted horn size of 7-year-olds represents the change, and upper and lower confidence limits of predicted horn size of 7-year-old males through time. Estimate and confidence intervals for environmental models represents the change, and upper and lower confidence limits of predicted horn size of 7-year-old males through time after the environment was accounted for. Estimate (β), p-value and R^2^ for selectivity analyses represents the relationship between predicted horn size (cm) of 7-year-old males and mean age of harvest of a cohort. Asterisks (*) on the beta coefficient indicate significance based on the 95% confidence interval for each analysis, with the exception of the selectivity analysis which was determined with p-value (α = 0.05). Environmental covariates indicate which covariate (if any) were included in the model to account for environmental effects.

| **State** | **Hunt Area** | **Measurement Type** | **Individual Linear Models** | | | | | **Individual Linear Models with Environment** | | | | | **Selectivity Analyses** | | | | **Environmental Covariates** |  |
| --- | --- | --- | --- | --- | --- | --- | --- | --- | --- | --- | --- | --- | --- | --- | --- | --- | --- | --- |
|  |  |  |  | | | **Confidence Interval** | |  | | | **Confidence Interval** | | |  | | |  | |
|  |  |  | **Est.** | **DF** | **R^2^** | **Lower** | **Upper** | **Est.** | **DF** | **R^2^** | **Lower** | **Upper** | | **Est.** | **R^2^** | ***p*- value** |  | |
| Alberta | AB-BowValley-Ghost-North | Length-base | -0.18* | 19 | 0.41 | -0.28 | -0.08 | -0.09 | 18 | 0.55 | -0.21 | 0.04 | | 0.06 | 0.03 | 0.44 | Early Life Precipitation | |
|  | AB-BowValley-Ghost-South | Length-base | -0.23* | 10 | 0.62 | -0.36 | -0.10 | -0.23* | 10 | 0.62 | -0.36 | -0.10 | | 0.02 | 0.01 | 0.81 |  | |
|  | AB-Cadomin | Length-base | -0.04 | 22 | 0.04 | -0.14 | 0.05 | -0.09 | 20 | 0.29 | -0.19 | 0.01 | | -0.06 | 0.03 | 0.42 | Early Life Amplitude Lifetime Amplitude | |
|  | AB-Clearwater-Ram | Length-base | -0.01 | 14 | <0.01 | -0.17 | 0.16 | -0.01 | 14 | <0.01 | -0.17 | 0.16 | | 0.06 | 0.02 | 0.64 |  | |
|  | AB-Clearwater-Ram-420 | Length-base | -0.13* | 22 | 0.31 | -0.22 | -0.04 | -0.08 | 21 | 0.43 | -0.18 | 0.01 | | -0.04 | 0.02 | 0.52 | Early Life Amplitude | |
|  | AB-Clearwater-Ram-422 | Length-base | -0.11* | 19 | 0.43 | -0.16 | -0.05 | -0.10* | 17 | 0.63 | -0.16 | -0.05 | | -0.25 | 0.20 | 0.04 | Early Life Amplitude Lifetime Amplitude | |
|  | AB-Clearwater-Ram-South | Length-base | -0.14* | 20 | 0.29 | -0.25 | -0.04 | -0.14* | 20 | 0.29 | -0.25 | -0.04 | | -0.02 | <0.01 | 0.81 |  | |
|  | AB-Kananaskis-North | Length-base | -0.11* | 22 | 0.23 | -0.19 | -0.02 | 0.02 | 20 | 0.50 | -0.09 | 0.14 | | -0.05 | 0.02 | 0.56 | Early Life Amplitude Lifetime integrated NDVI | |
|  | AB-Kananaskis-South | Length-base | -0.12* | 20 | 0.39 | -0.18 | -0.05 | -0.11* | 19 | 0.49 | -0.17 | -0.04 | | -0.03 | 0.01 | 0.74 |  | |
|  | AB-Livingstone-North | Length-base | -0.02 | 9 | 0.01 | -0.13 | 0.10 | -0.02 | 9 | 0.01 | -0.13 | 0.10 | | -0.03 | <0.01 | 0.85 |  | |
|  | AB-Nordegg-Chungo-North | Length-base | -0.12* | 22 | 0.34 | -0.19 | -0.04 | -0.12* | 22 | 0.34 | -0.19 | -0.04 | | -0.08 | 0.08 | 0.17 |  | |
|  | AB-Nordegg-Chungo-South | Length-base | -0.04 | 11 | 0.05 | -0.15 | 0.07 | -0.06 | 10 | 0.44 | -0.15 | 0.03 | | 0.13 | 0.10 | 0.29 | Gestation Snow Water Equivalent | |
|  | AB-Torrens | Length-base | 0.07 | 18 | 0.09 | -0.04 | 0.17 | 0.07 | 18 | 0.09 | -0.04 | 0.17 | | -0.29 | 0.29 | 0.02 |  | |
|  | AB-Westcastle-Yarrow | Length-base | 0.07 | 10 | 0.11 | -0.07 | 0.20 | 0.03 | 9 | 0.43 | -0.09 | 0.15 | | 0.29 | 0.16 | 0.20 | Gestation Minimum Temperature | |
|  | AB-Willmore-North | Length-base | -0.09* | 22 | 0.26 | -0.16 | -0.02 | -0.09* | 20 | 0.48 | -0.15 | -0.03 | | -0.06 | 0.04 | 0.37 | Gestation Snow Water Equivalent Lifetime integrated NDVI | |
|  | AB-Willmore-South | Length-base | -0.09* | 21 | 0.20 | -0.18 | -0.01 | -0.15* | 18 | 0.58 | -0.24 | -0.06 | | -0.13 | 0.36 | <0.01 | Early Life Amplitude Gestation Minimum Temperature Gestation Lifetime Amplitude | |
| Arizona | AZ-12.13 | Length-base | -0.06 | 10 | 0.06 | -0.21 | 0.10 | -0.06 | 10 | 0.06 | -0.21 | 0.10 | | -0.16 | 0.20 | 0.15 |  | |
|  | AZ-15.16 | Length-base | -0.12* | 19 | 0.22 | -0.23 | -0.01 | -0.12* | 16 | 0.65 | -0.22 | -0.03 | | 0.03 | 0.04 | 0.39 | Early Life Precipitation Early Life Snow Water Equivalent Lifetime Precipitation | |
|  | AZ-37.39.33.32.31 | Length-base | <0.01 | 11 | <0.01 | -0.14 | 0.14 | 0.02 | 10 | 0.30 | -0.11 | 0.15 | | 0.22 | 0.41 | 0.02 | Gestation Minimum Temperature | |
|  | AZ-40.46 | Length-base | -0.06 | 16 | 0.08 | -0.17 | 0.05 | 0.09 | 13 | 0.66 | -0.03 | 0.20 | | 0.13 | 0.12 | 0.17 | Gestation Snow Water Equivalent Lifetime Minimum Temperature Lifetime Precipitation | |
|  | AZ-44 | Length-base | 0.14* | 16 | 0.39 | 0.05 | 0.23 | 0.17* | 15 | 0.52 | 0.08 | 0.26 | | 0.02 | <0.01 | 0.83 | Lifetime Precipitation | |
|  | AZ-6.22.24 | Length-base | 0.12 | 10 | 0.14 | -0.09 | 0.34 | 0.12 | 10 | 0.14 | -0.09 | 0.34 | | 0.09 | 0.09 | 0.35 |  | |
| Colorado | CO-S01.18.19.37.40.57.58.73 | Length-base | -0.02 | 10 | 0.01 | -0.16 | 0.12 | -0.02 | 10 | 0.01 | -0.16 | 0.12 | | -0.54 | 0.32 | 0.05 |  | |
|  | CO-S02.59.67.74.77 | Length-base | 0.01 | 10 | <0.01 | -0.14 | 0.15 | 0.01 | 10 | <0.01 | -0.14 | 0.15 | | -0.02 | <0.01 | 0.91 |  | |
|  | CO-S03.04.23.27.32.41 | Length-base | -0.23* | 20 | 0.65 | -0.31 | -0.15 | -0.27* | 18 | 0.78 | -0.34 | -0.20 | | -0.13 | 0.13 | 0.10 | Early Life Amplitude Early Life Precipitation | |
|  | CO-S05.06.34.46.60.72 | Length-base | -0.20* | 18 | 0.46 | -0.30 | -0.09 | -0.20* | 18 | 0.46 | -0.30 | -0.09 | | -0.13 | 0.09 | 0.20 |  | |
|  | CO-S07.49.68 | Length-base | 0.01 | 7 | <0.01 | -0.28 | 0.29 | -0.04 | 6 | 0.28 | -0.32 | 0.25 | | 0.12 | 0.08 | 0.45 | Early Life Amplitude | |
|  | CO-S08.09.35.50 | Length-base | -0.11 | 19 | 0.14 | -0.23 | 0.02 | -0.05 | 17 | 0.43 | -0.18 | 0.07 | | 0.07 | 0.05 | 0.32 | Early Life Precipitation Lifetime Amplitude | |
|  | CO-S10.20.55 | Length-base | -0.07 | 10 | 0.11 | -0.22 | 0.07 | -0.07 | 10 | 0.11 | -0.22 | 0.07 | | -0.01 | <0.01 | 0.96 |  | |
|  | CO-S11.12.17.39.47.66 | Length-base | -0.07 | 19 | 0.12 | -0.16 | 0.02 | -0.04 | 17 | 0.41 | -0.12 | 0.04 | | -0.28 | 0.27 | 0.02 | Early Life Precipitation Gestation Minimum Temperature | |
|  | CO-S13.25.44 | Length-base | 0.21* | 14 | 0.62 | 0.12 | 0.31 | 0.21* | 14 | 0.62 | 0.12 | 0.31 | | 0.29 | 0.20 | 0.08 |  | |
|  | CO-S15.16.28.71 | Length-base | 0.15* | 13 | 0.47 | 0.05 | 0.24 | 0.15* | 13 | 0.47 | 0.05 | 0.24 | | 0.14 | 0.08 | 0.29 |  | |
|  | CO-S21.22.33.36.52.53 | Length-base | 0.11* | 11 | 0.47 | 0.03 | 0.19 | 0.26* | 9 | 0.82 | 0.16 | 0.36 | | 0.97 | 0.38 | 0.02 | Early Life Precipitation Lifetime integrated NDVI | |
|  | CO-S26.54.69.70 | Length-base | 0.02 | 12 | 0.01 | -0.16 | 0.21 | -0.11 | 10 | 0.53 | -0.28 | 0.06 | | -0.14 | 0.05 | 0.42 | Gestation Minimum Temperature Lifetime integrated NDVI | |
|  | CO-S29.30.31 | Length-base | 0.01 | 9 | <0.01 | -0.19 | 0.21 | 0.01 | 9 | <0.01 | -0.19 | 0.21 | | -0.05 | 0.01 | 0.82 |  | |
|  | CO-S38.48.61 | Length-base | 0.03 | 12 | 0.02 | -0.13 | 0.20 | 0.03 | 12 | 0.02 | -0.13 | 0.20 | | -0.20 | 0.16 | 0.16 |  | |
|  | CO-S51.65 | Length-base | 0.01 | 12 | <0.01 | -0.16 | 0.18 | -0.09 | 11 | 0.48 | -0.23 | 0.06 | | -0.17 | 0.13 | 0.21 | Early Life Amplitude | |
| Idaho | ID-17.21.21A.28 | Length-base | -0.19* | 8 | 0.41 | -0.37 | -0.01 | -0.19* | 8 | 0.41 | -0.37 | -0.01 | | -0.04 | 0.03 | 0.65 |  | |
|  | ID-19.20.20A.23.24.25 | Length-base | -0.12 | 13 | 0.23 | -0.26 | 0.01 | -0.12 | 13 | 0.23 | -0.26 | 0.01 | | -0.03 | 0.01 | 0.77 |  | |
|  | ID-26 | Length-base | -0.02 | 13 | 0.01 | -0.13 | 0.09 | -0.02 | 13 | 0.01 | -0.13 | 0.09 | | -0.02 | <0.01 | 0.90 |  | |
|  | ID-36.36A.36B.50 | Length-base | -0.08 | 13 | 0.08 | -0.23 | 0.08 | 0.11 | 11 | 0.50 | -0.08 | 0.29 | | -0.11 | 0.10 | 0.24 | Early Life Amplitude Lifetime Amplitude | |
|  | ID-37.37A.51 | Length-base | -0.01 | 8 | <0.01 | -0.17 | 0.16 | -0.01 | 8 | <0.01 | -0.17 | 0.16 | | -0.45 | 0.29 | 0.11 |  | |
|  | ID-40.41.42.46.47 | Length-base | 0.01 | 18 | <0.01 | -0.08 | 0.09 | 0.01 | 18 | <0.01 | -0.08 | 0.09 | | 0.10 | 0.05 | 0.36 |  | |
| Montana | MT-122.124.121.123.100.102.101 | Length-base | -0.09 | 7 | 0.20 | -0.25 | 0.07 | -0.08 | 6 | 0.14 | -0.28 | 0.12 | | 0.09 | 0.03 | 0.68 | Early Life Precipitation | |
|  | MT-210.212.213.261.250.270.340 | Length-base | -0.20 | 11 | 0.27 | -0.41 | 0.02 | -0.20 | 11 | 0.27 | -0.41 | 0.02 | | -0.35 | 0.40 | 0.02 |  | |
| New Mexico | NM-North | Full | -0.24* | 14 | 0.53 | -0.37 | -0.11 | -0.24* | 14 | 0.53 | -0.37 | -0.11 | | -0.24 | 0.19 | 0.09 |  | |
| Nevada | NV-12.14.32.33.34 | Full | 0.37* | 17 | 0.50 | 0.18 | 0.56 | 0.37* | 17 | 0.50 | 0.18 | 0.56 | | -0.01 | <0.01 | 0.86 |  | |
|  | NV-161.162.163.173 | Full | 0.06 | 14 | 0.03 | -0.12 | 0.23 | -0.11 | 12 | 0.62 | -0.29 | 0.07 | | 0.09 | 0.15 | 0.14 | Gestation Minimum Temperature Lifetime Minimum Temperature | |
|  | NV-202.204.205 | Full | 0.09 | 9 | 0.02 | -0.36 | 0.53 | 0.09 | 9 | 0.02 | -0.36 | 0.53 | | 0.01 | <0.01 | 0.89 |  | |
|  | NV-206.207.208.211.213.212 | Full | -0.02 | 15 | <0.01 | -0.21 | 0.17 | -0.02 | 15 | <0.01 | -0.21 | 0.17 | | 0.03 | <0.01 | 0.79 |  | |
|  | NV-244.268.286 | Full | -0.19 | 20 | 0.14 | -0.41 | 0.03 | -0.52* | 16 | 0.80 | -0.74 | -0.30 | | -0.01 | 0.01 | 0.72 | Early Life Snow Water Equivalent Gestation Minimum Temperature Gestation Snow Water Equivalent Lifetime Minimum Temperature | |
|  | NV-252.253.254 | Full | 0.30 | 10 | 0.29 | -0.03 | 0.64 | 0.30 | 10 | 0.29 | -0.03 | 0.64 | | 0.06 | 0.09 | 0.36 |  | |
|  | NV-261.262 | Full | 0.01 | 15 | <0.01 | -0.18 | 0.20 | 0.01 | 15 | <0.01 | -0.18 | 0.20 | | 0.02 | 0.02 | 0.57 |  | |
|  | NV-263.264.265.266 | Full | 0.24* | 13 | 0.39 | 0.06 | 0.42 | 0.24* | 13 | 0.39 | 0.06 | 0.42 | | -0.05 | 0.07 | 0.32 |  | |
|  | NV-271.267.272.243 | Full | -0.12 | 19 | 0.05 | -0.35 | 0.12 | 0.13 | 17 | 0.49 | -0.12 | 0.37 | | 0.05 | 0.14 | 0.10 | Lifetime Minimum Temperature Lifetime Precipitation | |
|  | NV-280.281.282.283.284 | Full | -0.07 | 17 | 0.02 | -0.32 | 0.18 | -0.15 | 15 | 0.69 | -0.34 | 0.03 | | -0.05 | 0.05 | 0.35 | Gestation Minimum Temperature Lifetime Precipitation | |
|  | NV-31.35.51 | Full | -0.18 | 16 | 0.17 | -0.38 | 0.03 | -0.18 | 16 | 0.17 | -0.38 | 0.03 | | 0.12 | 0.18 | 0.08 |  | |
|  | NV-44.45.153.181.182.183.184 | Full | 0.09 | 11 | 0.19 | -0.03 | 0.21 | 0.09 | 11 | 0.19 | -0.03 | 0.21 | | -0.05 | 0.02 | 0.67 |  | |
| Oregon | OR-Deschutes | Full | 0.08 | 7 | 0.02 | -0.39 | 0.55 | 0.22 | 6 | 0.52 | -0.12 | 0.56 | | -0.05 | 0.01 | 0.78 | Gestation Snow Water Equivalent | |
|  | OR-GrandeRonde | Full | -0.23* | 14 | 0.25 | -0.46 | <0.01 | -0.18 | 12 | 0.70 | -0.39 | 0.03 | | <0.01 | <0.01 | 0.92 | Gestation Minimum Temperature Gestation Snow Water Equivalent | |
|  | OR-JohnDay | Full | 0.10 | 11 | 0.04 | -0.22 | 0.41 | 0.10 | 11 | 0.04 | -0.22 | 0.41 | | 0.09 | 0.10 | 0.30 |  | |
|  | OR-Klamath | Full | -0.23* | 22 | 0.36 | -0.36 | -0.09 | -0.26* | 19 | 0.77 | -0.39 | -0.14 | | -0.03 | 0.02 | 0.49 | Early Life Precipitation Lifetime Amplitude Lifetime integrated NDVI | |
|  | OR-Malheur | Full | -0.14 | 21 | 0.12 | -0.31 | 0.03 | -0.14 | 21 | 0.12 | -0.31 | 0.03 | | 0.01 | <0.01 | 0.92 |  | |
| Utah | UT-12.13.14.15.26 | Length-base | -0.28 | 11 | 0.28 | -0.57 | 0.02 | -0.08 | 10 | 0.51 | -0.41 | 0.25 | | -0.31 | 0.11 | 0.26 | Lifetime Precipitation | |
|  | UT-8.10.11 | Length-base | <0.01 | 10 | <0.01 | -0.16 | 0.16 | <0.01 | 10 | <0.01 | -0.16 | 0.16 | | 0.17 | 0.05 | 0.48 |  | |
| Wyoming | WY-ClarksForkTroutPeak | Length-base | 0.05 | 22 | 0.04 | -0.06 | 0.16 | -0.11 | 19 | 0.71 | -0.26 | 0.05 | | -0.02 | <0.01 | 0.81 | Early Life Precipitation Gestation Minimum Temperature Lifetime integrated NDVI | |
|  | WY-FrancsPeak | Length-base | -0.08* | 22 | 0.20 | -0.16 | -0.01 | -0.08* | 22 | 0.20 | -0.16 | -0.01 | | 0.08 | 0.09 | 0.16 |  | |
|  | WY-Laramie | Length-base | 0.16 | 14 | 0.14 | -0.07 | 0.40 | 0.12 | 11 | 0.73 | -0.08 | 0.32 | | -0.01 | <0.01 | 0.95 | Early Life Amplitude Early Life Precipitation Gestation Minimum Temperature | |
|  | WY-TargheeDarbyJackson | Length-base | -0.01 | 15 | <0.01 | -0.16 | 0.14 | -0.01 | 15 | <0.01 | -0.16 | 0.14 | | 0.12 | 0.05 | 0.37 |  | |
|  | WY-WaipitiRidge | Length-base | -0.04 | 22 | 0.05 | -0.13 | 0.04 | -0.16* | 19 | 0.40 | -0.30 | -0.02 | | 0.15 | 0.08 | 0.18 | Early Life Amplitude Early Life Precipitation Gestation Snow Water Equivalent | |
|  | WY-WhiskeyMtn | Length-base | -0.05 | 21 | 0.09 | -0.13 | 0.02 | -0.05 | 21 | 0.09 | -0.13 | 0.02 | | <0.01 | <0.01 | 1.00 |  | |
|  | WY-YontsPeak | Length-base | 0.03 | 20 | 0.02 | -0.06 | 0.12 | 0.03 | 20 | 0.02 | -0.06 | 0.12 | | 0.01 | <0.01 | 0.94 |  | |

**Appendix S2. Correlation between horn measurements.**

To evaluate the validity of using two different metrics of horn size (full score and length-base score), we looked at the correlation between the length-base score and the full score of all records provided by state agencies with individual measurements. Using 3,038 records from four states in the U.S. (Arizona, North Dakota, New Mexico, and Utah), we assessed the correlation between the length-base score and full score. We found that across subspecies and states, the two scores were highly correlated (r = 0.94).

**Appendix S3. Assessment of simulated harvest records.**

To evaluate if our modeling approach would reveal changes in the horn growth of males, we assessed the effects of harvest in 180 simulated hunt areas over a 35-year period. For each simulation, we built an individual-based model for populations of male bighorn sheep using parameters adapted from Festa-Bianchet et al. (2015). We developed the model using program R.

**Horn Growth**

Parameters describing annual growth of horns are listed in Table S4. We developed the starting parameters for horn size and growth based on the starting parameters from the simulations in Festa-Bianchet et al. (2015), and then adjusted growth increments using harvest data for mountain sheep from across western North America to develop horn sizes that were comparable to horn sizes of harvested mountain sheep across the range of our dataset.

We modeled horn growth as either stable, increasing, or decreasing over time within a simulated population. Stable horn growth remained constant over the simulation period. Decreasing horn growth was modeled as a decline of 0.1% in the horn size of recruited males (males of age 2) each year over the simulation period. Increasing horn growth was modeled as an increase of 0.1% in the horn size of recruited males (males of age 2) each year over the simulation period.

**Model**

We parameterized an individual-based model in program R (3.5.1) that we adapted from a previously published model of horn growth and harvest for a population of bighorn sheep (Festa-Bianchet et al. 2015). We recruited animals into the simulated population at age 2, and did not allow animals to live past age 14. At recruitment, 400 males (SD=30) entered the population with a horn length of 30.5 cm (SD=4.9) and a base circumference of 21.1 cm (SD=2.8).

Each year, an animal’s horn size was determined by adding on annual growth (± standard deviation) to its previous horn size (Table S4). Annual growth was determined by an increase in both horn length and horn circumference. We determined age-specific survival rates of males in the population using survival estimates in the model described by Festa-Bianchet et al. (2015; Table S3). Within each year, we determined natural mortality of each animal based on the probability of survival for each individual age class. Animals that survived a given year remained in the population, became 1 year older, and their horns grew.

To simulate each of the hunt areas, we first developed a huntable population using a 15-year burn-in period where there was no mortality from harvest (i.e., animals were recruited each year and natural mortality occurred, but there was no harvest of individuals). During the burn-in period, we modeled natural mortality (Table S3) and horn growth of individuals within each year (Table S4).

After 15 initial time steps (i.e., the burn-in period), all age classes were potentially represented, and we allowed mortality to occur via simulated harvest. Within the simulated populations, we modeled all combinations of the following harvest rates and selectivity: 1, 5, 10, and 20 percent harvest rates (i.e., 1%, 5%, 10% or 20% of animals were harvested from the population), and no selectivity, medium selectivity, and high selectivity. Harvest with no selectivity occurred when animals were randomly harvested from the population. Harvest with medium selectivity limited harvest to animals that had horn sizes above the 50^th^ percentile. Harvest with high selectivity limited harvest to animals that had horn sizes above the 75^th^ percentile. Within each time step of the simulation, we first modeled recruitment of new males into the population, followed by mortality from harvest, natural mortality, annual horn growth, and then aging of any surviving individuals.

For each combination of harvest rate and selectivity (e.g., 5% harvest, medium selectivity) we simulated populations that had increasing (n=5), decreasing (n=5), or stable (n=5) horn size over time. We modeled change for the populations with decreasing and increasing horn size as 0.1% each year. We chose a relatively small change in horn size because we wanted to evaluate if our models were capable of detecting changes in horn size that might occur very slowly over time. In total, with 12 potential combinations of harvest rates and selectivity and 3 different trajectories of horn growth, we evaluated 180 simulated hunt areas.

We simulated harvest rates of differing selectivity and intensity to evaluate how changes in horn size and growth that occur under different management systems might be detected by our analytical framework and the models that we used in the manuscript. We modeled horn growth curves of each cohort of harvested animals within the hunt area in a manner identical to the approach we used for empirical harvest data, and assessed if we were able to detect changes in horn growth over time. For this simulation exercise, we included the “length base” metric of horn size. We only evaluated changes in horn growth over the last 35 years of each simulated population because that matched the timeframe in which we evaluated changes in horn growth with the harvest data from state and provincial agencies.

We modeled horn growth curves using harvest data from the simulated populations and the same modeling technique as described in the main text. In addition to evaluating changes in horn size through the modeling efforts we used in our manuscript, we assessed changes in horn size of all 7-year-old males of each cohort in each population using linear regression. We used cohort year as the predictor variable and horn size as the response variable.

Of the 180 simulated populations, harvest data in 6 hunt areas (3.3%) resulted in detectable changes in horn growth of 7-year olds when horn size of 7-year olds within the population was stable over time (Table S6). For 1 of the 6 hunt areas where we detected change in horn growth, perhaps by random chance, average size of 7-year-olds within the population also changed. In the remaining 5 hunt areas, horn size of 7-year-olds in the population remained stable, but the predicted horn size of 7-year-old males increased (n=2) or decreased (n=3). In 100% of the hunt areas with increasing (n=60) or decreasing (n=60) horn size, predicted horn size of 7-year-old males based on harvest data also changed in the corresponding direction (Table S5, Table S6). Overall, our analytical approach was robust to detecting changes in horn growth, regardless of the degree of harvest intensity or selectivity and corresponding concerns associated with bias in harvest data.

Table S3. Probability of survival for each age class adapted from Festa-Bianchet et al. (2015).

| **Age** | **Probability of Survival** |
| --- | --- |
| 2 | 0.95 |
| 3 | 0.9 |
| 4 | 0.86 |
| 5 | 0.841 |
| 6 | 0.826 |
| 7 | 0.805 |
| 8 | 0.788 |
| 9 | 0.77 |
| 10 | 0.74 |
| 11 | 0.72 |
| 12 | 0.69 |
| 13 | 0.3 |
| 14 | 0 |

Table S4. Annual horn growth (cm) for bighorn sheep males by age class, adapted from Festa-Bianchet et al. (2015) and from harvest records of wild sheep in North America.

| Age | Length Increase | Length SD | Base Increase | Base SD |
| --- | --- | --- | --- | --- |
| 2 | 17.27 | 3.30 | 13.46 | 1.21 |
| 3 | 13.46 | 3.05 | 7.87 | 1.27 |
| 4 | 8.13 | 2.79 | 2.79 | 0.41 |
| 5 | 5.84 | 2.29 | 2.54 | 0.93 |
| 6 | 3.56 | 1.78 | 0.00 | 0.18 |
| 7 | 3.30 | 1.27 | 0.00 | 0.08 |
| 8 | 2.54 | 1.02 | 0.00 | 0.08 |
| 9 | 2.29 | 0.51 | 0.00 | 0.08 |
| 10 | 0.00 | 0.76 | 0.00 | 0.08 |
| 11 | 0.00 | 0.51 | 0.00 | 0.08 |
| 12 | 0.00 | 0.76 | 0.00 | 0.08 |
| 13 | 0.00 | 0.51 | 0.00 | 0.08 |
| 14 | 0.00 | 0.76 | 0.00 | 0.08 |

Table S5. Model output for each analysis for simulated hunt areas. Slope and p-value for predicted horn size of 7-year-olds represents the change and significance of predicted horn size of 7-year-old males through time. Slope and p-value for population 7-year-olds represents the change and significance of horn size of all 7-year-old males in a population through time. HR indicates harvest rate of males (1, 5, 10, or 20%). Selectivity indicates the degree of selection (low, medium or high). Trend represents direction of change that was implemented in the model (increasing, decreasing, or stable). Significance was determined with p-value (α = 0.05).

|  | **Predicted 7 Year Olds** | | | | **Population 7 Year Olds** | | | |  |  |
| --- | --- | --- | --- | --- | --- | --- | --- | --- | --- | --- |
| **Hunt Area ID** | **β** | **R^2^** | **P-Value** | **SE** | **β** | **R^2^** | **P-Value** | **SE** | **Selectivity** | **trend** |
| high1_0.25a | 0.25 | 0.61 | <0.01 | 0.04 | 0.26 | 0.92 | <0.01 | 0.01 | High | increasing |
| high1_0.25b | 0.29 | 0.59 | <0.01 | 0.04 | 0.26 | 0.86 | <0.01 | 0.02 | High | increasing |
| high1_0.25c | 0.28 | 0.63 | <0.01 | 0.04 | 0.23 | 0.92 | <0.01 | 0.01 | High | increasing |
| high1_0.25d | 0.26 | 0.54 | <0.01 | 0.04 | 0.21 | 0.9 | <0.01 | 0.01 | High | increasing |
| high1_0.25e | 0.32 | 0.71 | <0.01 | 0.04 | 0.26 | 0.92 | <0.01 | 0.01 | High | increasing |
| high10_0.25a | 0.26 | 0.89 | <0.01 | 0.02 | 0.28 | 0.89 | <0.01 | 0.02 | High | increasing |
| high10_0.25b | 0.25 | 0.87 | <0.01 | 0.02 | 0.24 | 0.79 | <0.01 | 0.02 | High | increasing |
| high10_0.25c | 0.28 | 0.91 | <0.01 | 0.02 | 0.26 | 0.86 | <0.01 | 0.02 | High | increasing |
| high10_0.25d | 0.29 | 0.92 | <0.01 | 0.02 | 0.24 | 0.87 | <0.01 | 0.02 | High | increasing |
| high10_0.25e | 0.25 | 0.89 | <0.01 | 0.02 | 0.24 | 0.83 | <0.01 | 0.02 | High | increasing |
| high20_0.25a | 0.20 | 0.81 | <0.01 | 0.02 | 0.17 | 0.12 | 0.04 | 0.08 | High | increasing |
| high20_0.25b | 0.26 | 0.75 | <0.01 | 0.03 | 0.29 | 0.39 | <0.01 | 0.06 | High | increasing |
| high20_0.25c | 0.23 | 0.7 | <0.01 | 0.03 | 0.32 | 0.35 | <0.01 | 0.08 | High | increasing |
| high20_0.25d | 0.22 | 0.65 | <0.01 | 0.03 | 0.26 | 0.25 | <0.01 | 0.08 | High | increasing |
| high20_0.25e | 0.23 | 0.76 | <0.01 | 0.02 | 0.13 | 0.14 | 0.03 | 0.06 | High | increasing |
| high5_0.25a | 0.26 | 0.89 | <0.01 | 0.02 | 0.27 | 0.9 | <0.01 | 0.02 | High | increasing |
| high5_0.25b | 0.31 | 0.85 | <0.01 | 0.02 | 0.27 | 0.91 | <0.01 | 0.01 | High | increasing |
| high5_0.25c | 0.26 | 0.83 | <0.01 | 0.02 | 0.25 | 0.85 | <0.01 | 0.02 | High | increasing |
| high5_0.25d | 0.31 | 0.9 | <0.01 | 0.02 | 0.27 | 0.91 | <0.01 | 0.01 | High | increasing |
| high5_0.25e | 0.25 | 0.81 | <0.01 | 0.02 | 0.25 | 0.89 | <0.01 | 0.02 | High | increasing |
| low1_0.25a | 0.32 | 0.43 | <0.01 | 0.07 | 0.29 | 0.91 | <0.01 | 0.02 | Low | increasing |
| low1_0.25b | 0.20 | 0.16 | 0.02 | 0.09 | 0.27 | 0.9 | <0.01 | 0.02 | Low | increasing |
| low1_0.25c | 0.26 | 0.38 | <0.01 | 0.06 | 0.25 | 0.87 | <0.01 | 0.02 | Low | increasing |
| low1_0.25d | 0.15 | 0.16 | 0.03 | 0.07 | 0.26 | 0.89 | <0.01 | 0.02 | Low | increasing |
| low1_0.25e | 0.29 | 0.38 | <0.01 | 0.07 | 0.28 | 0.95 | <0.01 | 0.01 | Low | increasing |
| low10_0.25a | 0.24 | 0.85 | <0.01 | 0.02 | 0.24 | 0.81 | <0.01 | 0.02 | Low | increasing |
| low10_0.25b | 0.25 | 0.69 | <0.01 | 0.03 | 0.26 | 0.86 | <0.01 | 0.02 | Low | increasing |
| low10_0.25c | 0.20 | 0.65 | <0.01 | 0.03 | 0.24 | 0.87 | <0.01 | 0.02 | Low | increasing |
| low10_0.25d | 0.26 | 0.76 | <0.01 | 0.03 | 0.28 | 0.87 | <0.01 | 0.02 | Low | increasing |
| low10_0.25e | 0.29 | 0.71 | <0.01 | 0.03 | 0.27 | 0.86 | <0.01 | 0.02 | Low | increasing |
| low20_0.25a | 0.28 | 0.79 | <0.01 | 0.03 | 0.24 | 0.69 | <0.01 | 0.03 | Low | increasing |
| low20_0.25b | 0.25 | 0.86 | <0.01 | 0.02 | 0.24 | 0.84 | <0.01 | 0.02 | Low | increasing |
| low20_0.25c | 0.25 | 0.8 | <0.01 | 0.02 | 0.22 | 0.75 | <0.01 | 0.02 | Low | increasing |
| low20_0.25d | 0.30 | 0.85 | <0.01 | 0.02 | 0.28 | 0.78 | <0.01 | 0.03 | Low | increasing |
| low20_0.25e | 0.19 | 0.59 | <0.01 | 0.03 | 0.23 | 0.76 | <0.01 | 0.02 | Low | increasing |
| low5_0.25a | 0.29 | 0.71 | <0.01 | 0.03 | 0.28 | 0.85 | <0.01 | 0.02 | Low | increasing |
| low5_0.25b | 0.29 | 0.7 | <0.01 | 0.03 | 0.28 | 0.91 | <0.01 | 0.02 | Low | increasing |
| low5_0.25c | 0.19 | 0.46 | <0.01 | 0.04 | 0.26 | 0.86 | <0.01 | 0.02 | Low | increasing |
| low5_0.25d | 0.26 | 0.7 | <0.01 | 0.03 | 0.26 | 0.92 | <0.01 | 0.01 | Low | increasing |
| low5_0.25e | 0.29 | 0.74 | <0.01 | 0.03 | 0.27 | 0.84 | <0.01 | 0.02 | Low | increasing |
| med1_0.25a | 0.24 | 0.34 | <0.01 | 0.06 | 0.28 | 0.91 | <0.01 | 0.02 | Medium | increasing |
| med1_0.25b | 0.25 | 0.46 | <0.01 | 0.05 | 0.26 | 0.89 | <0.01 | 0.02 | Medium | increasing |
| med1_0.25c | 0.22 | 0.49 | <0.01 | 0.04 | 0.26 | 0.92 | <0.01 | 0.01 | Medium | increasing |
| med1_0.25d | 0.34 | 0.66 | <0.01 | 0.04 | 0.27 | 0.86 | <0.01 | 0.02 | Medium | increasing |
| med1_0.25e | 0.18 | 0.25 | <0.01 | 0.06 | 0.25 | 0.89 | <0.01 | 0.02 | Medium | increasing |
| med10_0.25a | 0.25 | 0.79 | <0.01 | 0.02 | 0.29 | 0.85 | <0.01 | 0.02 | Medium | increasing |
| med10_0.25b | 0.23 | 0.73 | <0.01 | 0.03 | 0.25 | 0.9 | <0.01 | 0.01 | Medium | increasing |
| med10_0.25c | 0.26 | 0.76 | <0.01 | 0.03 | 0.23 | 0.8 | <0.01 | 0.02 | Medium | increasing |
| med10_0.25d | 0.24 | 0.8 | <0.01 | 0.02 | 0.23 | 0.84 | <0.01 | 0.02 | Medium | increasing |
| med10_0.25e | 0.24 | 0.76 | <0.01 | 0.02 | 0.24 | 0.8 | <0.01 | 0.02 | Medium | increasing |
| med20_0.25a | 0.20 | 0.62 | <0.01 | 0.03 | 0.19 | 0.44 | <0.01 | 0.04 | Medium | increasing |
| med20_0.25b | 0.26 | 0.79 | <0.01 | 0.02 | 0.28 | 0.65 | <0.01 | 0.04 | Medium | increasing |
| med20_0.25c | 0.26 | 0.75 | <0.01 | 0.03 | 0.23 | 0.56 | <0.01 | 0.04 | Medium | increasing |
| med20_0.25d | 0.28 | 0.85 | <0.01 | 0.02 | 0.26 | 0.65 | <0.01 | 0.03 | Medium | increasing |
| med20_0.25e | 0.24 | 0.74 | <0.01 | 0.03 | 0.30 | 0.8 | <0.01 | 0.03 | Medium | increasing |
| med5_0.25a | 0.31 | 0.79 | <0.01 | 0.03 | 0.27 | 0.9 | <0.01 | 0.02 | Medium | increasing |
| med5_0.25b | 0.20 | 0.78 | <0.01 | 0.02 | 0.25 | 0.88 | <0.01 | 0.02 | Medium | increasing |
| med5_0.25c | 0.24 | 0.73 | <0.01 | 0.03 | 0.23 | 0.82 | <0.01 | 0.02 | Medium | increasing |
| med5_0.25d | 0.27 | 0.72 | <0.01 | 0.03 | 0.26 | 0.9 | <0.01 | 0.01 | Medium | increasing |
| med5_0.25e | 0.27 | 0.66 | <0.01 | 0.04 | 0.27 | 0.88 | <0.01 | 0.02 | Medium | increasing |
| high1_-0.25a | -0.30 | 0.7 | <0.01 | 0.04 | -0.28 | 0.94 | <0.01 | 0.01 | High | decreasing |
| high1_-0.25b | -0.25 | 0.52 | <0.01 | 0.04 | -0.28 | 0.94 | <0.01 | 0.01 | High | decreasing |
| high1_-0.25c | -0.23 | 0.54 | <0.01 | 0.04 | -0.27 | 0.91 | <0.01 | 0.01 | High | decreasing |
| high1_-0.25d | -0.26 | 0.64 | <0.01 | 0.04 | -0.25 | 0.94 | <0.01 | 0.01 | High | decreasing |
| high1_-0.25e | -0.23 | 0.62 | <0.01 | 0.03 | -0.27 | 0.91 | <0.01 | 0.01 | High | decreasing |
| high10_-0.25a | -0.28 | 0.91 | <0.01 | 0.02 | -0.27 | 0.91 | <0.01 | 0.01 | High | decreasing |
| high10_-0.25b | -0.27 | 0.88 | <0.01 | 0.02 | -0.27 | 0.87 | <0.01 | 0.02 | High | decreasing |
| high10_-0.25c | -0.25 | 0.88 | <0.01 | 0.02 | -0.25 | 0.88 | <0.01 | 0.02 | High | decreasing |
| high10_-0.25d | -0.26 | 0.83 | <0.01 | 0.02 | -0.27 | 0.92 | <0.01 | 0.01 | High | decreasing |
| high10_-0.25e | -0.25 | 0.88 | <0.01 | 0.02 | -0.26 | 0.91 | <0.01 | 0.01 | High | decreasing |
| high20_-0.25a | -0.25 | 0.71 | <0.01 | 0.03 | -0.23 | 0.22 | <0.01 | 0.07 | High | decreasing |
| high20_-0.25b | -0.25 | 0.84 | <0.01 | 0.02 | -0.21 | 0.31 | <0.01 | 0.05 | High | decreasing |
| high20_-0.25c | -0.24 | 0.72 | <0.01 | 0.03 | -0.28 | 0.37 | <0.01 | 0.06 | High | decreasing |
| high20_-0.25d | -0.30 | 0.86 | <0.01 | 0.02 | -0.26 | 0.3 | <0.01 | 0.07 | High | decreasing |
| high20_-0.25e | -0.22 | 0.81 | <0.01 | 0.02 | -0.12 | 0.11 | 0.05 | 0.06 | High | decreasing |
| high5_-0.25a | -0.27 | 0.86 | <0.01 | 0.02 | -0.24 | 0.9 | <0.01 | 0.01 | High | decreasing |
| high5_-0.25b | -0.28 | 0.89 | <0.01 | 0.02 | -0.26 | 0.89 | <0.01 | 0.02 | High | decreasing |
| high5_-0.25c | -0.27 | 0.87 | <0.01 | 0.02 | -0.24 | 0.89 | <0.01 | 0.01 | High | decreasing |
| high5_-0.25d | -0.23 | 0.85 | <0.01 | 0.02 | -0.25 | 0.91 | <0.01 | 0.01 | High | decreasing |
| high5_-0.25e | -0.28 | 0.92 | <0.01 | 0.01 | -0.24 | 0.93 | <0.01 | 0.01 | High | decreasing |
| low1_-0.25a | -0.18 | 0.21 | 0.01 | 0.06 | -0.26 | 0.91 | <0.01 | 0.01 | Low | decreasing |
| low1_-0.25b | -0.25 | 0.32 | <0.01 | 0.07 | -0.27 | 0.92 | <0.01 | 0.01 | Low | decreasing |
| low1_-0.25c | -0.28 | 0.41 | <0.01 | 0.06 | -0.25 | 0.91 | <0.01 | 0.01 | Low | decreasing |
| low1_-0.25d | -0.27 | 0.41 | <0.01 | 0.06 | -0.26 | 0.9 | <0.01 | 0.02 | Low | decreasing |
| low1_-0.25e | -0.25 | 0.38 | <0.01 | 0.06 | -0.26 | 0.9 | <0.01 | 0.01 | Low | decreasing |
| low10_-0.25a | -0.26 | 0.85 | <0.01 | 0.02 | -0.28 | 0.9 | <0.01 | 0.02 | Low | decreasing |
| low10_-0.25b | -0.25 | 0.79 | <0.01 | 0.02 | -0.28 | 0.88 | <0.01 | 0.02 | Low | decreasing |
| low10_-0.25c | -0.25 | 0.8 | <0.01 | 0.02 | -0.23 | 0.87 | <0.01 | 0.02 | Low | decreasing |
| low10_-0.25d | -0.28 | 0.88 | <0.01 | 0.02 | -0.24 | 0.92 | <0.01 | 0.01 | Low | decreasing |
| low10_-0.25e | -0.28 | 0.75 | <0.01 | 0.03 | -0.28 | 0.84 | <0.01 | 0.02 | Low | decreasing |
| low20_-0.25a | -0.26 | 0.83 | <0.01 | 0.02 | -0.22 | 0.75 | <0.01 | 0.02 | Low | decreasing |
| low20_-0.25b | -0.25 | 0.74 | <0.01 | 0.03 | -0.24 | 0.79 | <0.01 | 0.02 | Low | decreasing |
| low20_-0.25c | -0.26 | 0.83 | <0.01 | 0.02 | -0.28 | 0.8 | <0.01 | 0.02 | Low | decreasing |
| low20_-0.25d | -0.25 | 0.83 | <0.01 | 0.02 | -0.22 | 0.73 | <0.01 | 0.02 | Low | decreasing |
| low20_-0.25e | -0.23 | 0.77 | <0.01 | 0.02 | -0.24 | 0.71 | <0.01 | 0.03 | Low | decreasing |
| low5_-0.25a | -0.27 | 0.71 | <0.01 | 0.03 | -0.26 | 0.89 | <0.01 | 0.02 | Low | decreasing |
| low5_-0.25b | -0.25 | 0.72 | <0.01 | 0.03 | -0.26 | 0.89 | <0.01 | 0.02 | Low | decreasing |
| low5_-0.25c | -0.27 | 0.69 | <0.01 | 0.03 | -0.28 | 0.86 | <0.01 | 0.02 | Low | decreasing |
| low5_-0.25d | -0.28 | 0.76 | <0.01 | 0.03 | -0.28 | 0.93 | <0.01 | 0.01 | Low | decreasing |
| low5_-0.25e | -0.25 | 0.7 | <0.01 | 0.03 | -0.23 | 0.91 | <0.01 | 0.01 | Low | decreasing |
| med1_-0.25a | -0.22 | 0.37 | <0.01 | 0.05 | -0.27 | 0.93 | <0.01 | 0.01 | Medium | decreasing |
| med1_-0.25b | -0.26 | 0.56 | <0.01 | 0.04 | -0.27 | 0.96 | <0.01 | 0.01 | Medium | decreasing |
| med1_-0.25c | -0.14 | 0.28 | <0.01 | 0.04 | -0.23 | 0.84 | <0.01 | 0.02 | Medium | decreasing |
| med1_-0.25d | -0.22 | 0.38 | <0.01 | 0.05 | -0.24 | 0.91 | <0.01 | 0.01 | Medium | decreasing |
| med1_-0.25e | -0.19 | 0.44 | <0.01 | 0.04 | -0.27 | 0.91 | <0.01 | 0.01 | Medium | decreasing |
| med10_-0.25a | -0.27 | 0.83 | <0.01 | 0.02 | -0.25 | 0.82 | <0.01 | 0.02 | Medium | decreasing |
| med10_-0.25b | -0.27 | 0.84 | <0.01 | 0.02 | -0.25 | 0.88 | <0.01 | 0.02 | Medium | decreasing |
| med10_-0.25c | -0.24 | 0.81 | <0.01 | 0.02 | -0.24 | 0.89 | <0.01 | 0.01 | Medium | decreasing |
| med10_-0.25d | -0.27 | 0.84 | <0.01 | 0.02 | -0.26 | 0.94 | <0.01 | 0.01 | Medium | decreasing |
| med10_-0.25e | -0.25 | 0.8 | <0.01 | 0.02 | -0.27 | 0.85 | <0.01 | 0.02 | Medium | decreasing |
| med20_-0.25a | -0.25 | 0.8 | <0.01 | 0.02 | -0.24 | 0.68 | <0.01 | 0.03 | Medium | decreasing |
| med20_-0.25b | -0.23 | 0.74 | <0.01 | 0.02 | -0.26 | 0.64 | <0.01 | 0.03 | Medium | decreasing |
| med20_-0.25c | -0.23 | 0.77 | <0.01 | 0.02 | -0.19 | 0.51 | <0.01 | 0.03 | Medium | decreasing |
| med20_-0.25d | -0.25 | 0.83 | <0.01 | 0.02 | -0.22 | 0.6 | <0.01 | 0.03 | Medium | decreasing |
| med20_-0.25e | -0.23 | 0.84 | <0.01 | 0.02 | -0.24 | 0.66 | <0.01 | 0.03 | Medium | decreasing |
| med5_-0.25a | -0.25 | 0.72 | <0.01 | 0.03 | -0.24 | 0.88 | <0.01 | 0.02 | Medium | decreasing |
| med5_-0.25b | -0.27 | 0.81 | <0.01 | 0.02 | -0.27 | 0.93 | <0.01 | 0.01 | Medium | decreasing |
| med5_-0.25c | -0.25 | 0.82 | <0.01 | 0.02 | -0.26 | 0.9 | <0.01 | 0.02 | Medium | decreasing |
| med5_-0.25d | -0.27 | 0.79 | <0.01 | 0.02 | -0.23 | 0.87 | <0.01 | 0.02 | Medium | decreasing |
| med5_-0.25e | -0.22 | 0.7 | <0.01 | 0.03 | -0.28 | 0.93 | <0.01 | 0.01 | Medium | decreasing |
| high1_0a | 0.02 | 0.01 | 0.61 | 0.04 | -0.01 | 0.03 | 0.35 | 0.01 | High | stable |
| high1_0b | 0.01 | <0.01 | 0.86 | 0.04 | 0.02 | 0.1 | 0.06 | 0.01 | High | stable |
| high1_0c | 0.04 | 0.04 | 0.3 | 0.04 | 0.01 | 0.01 | 0.59 | 0.01 | High | stable |
| high1_0d | -0.01 | 0.01 | 0.69 | 0.03 | 0.03 | 0.11 | 0.05 | 0.02 | High | stable |
| high1_0e | -0.02 | 0.01 | 0.69 | 0.04 | -0.01 | 0.01 | 0.51 | 0.01 | High | stable |
| high10_0a | 0.01 | <0.01 | 0.71 | 0.02 | 0.02 | 0.02 | 0.38 | 0.02 | High | stable |
| high10_0b | 0.01 | 0.02 | 0.41 | 0.02 | <0.01 | <0.01 | 0.96 | 0.02 | High | stable |
| high10_0c | 0.02 | 0.04 | 0.28 | 0.02 | <0.01 | <0.01 | 0.8 | 0.02 | High | stable |
| high10_0d | -0.01 | 0.01 | 0.6 | 0.02 | -0.01 | 0.02 | 0.42 | 0.01 | High | stable |
| high10_0e | -0.01 | 0.01 | 0.54 | 0.01 | -0.02 | 0.03 | 0.32 | 0.02 | High | stable |
| high20_0a | 0.01 | 0.01 | 0.64 | 0.02 | 0.07 | 0.04 | 0.27 | 0.06 | High | stable |
| high20_0b | 0.04 | 0.09 | 0.1 | 0.02 | 0.07 | 0.04 | 0.26 | 0.06 | High | stable |
| high20_0c | 0.03 | 0.04 | 0.26 | 0.02 | 0.01 | <0.01 | 0.93 | 0.07 | High | stable |
| high20_0d | 0.02 | 0.02 | 0.41 | 0.03 | -0.05 | 0.02 | 0.48 | 0.08 | High | stable |
| high20_0e | 0.02 | 0.05 | 0.24 | 0.02 | 0.06 | 0.03 | 0.35 | 0.06 | High | stable |
| high5_0a | -0.05 | 0.19 | 0.01 | 0.02 | -0.04 | 0.24 | <0.01 | 0.01 | High | stable |
| high5_0b | 0.02 | 0.04 | 0.3 | 0.02 | <0.01 | <0.01 | 0.8 | 0.01 | High | stable |
| high5_0c | <0.01 | <0.01 | 0.96 | 0.03 | <0.01 | <0.01 | 0.94 | 0.02 | High | stable |
| high5_0d | -0.01 | 0.01 | 0.6 | 0.02 | <0.01 | <0.01 | 0.82 | 0.01 | High | stable |
| high5_0e | 0.01 | 0.01 | 0.54 | 0.02 | 0.01 | 0.01 | 0.54 | 0.02 | High | stable |
| low1_0a | 0.05 | 0.03 | 0.32 | 0.05 | <0.01 | <0.01 | 0.8 | 0.01 | Low | stable |
| low1_0b | -0.07 | 0.03 | 0.38 | 0.08 | 0.02 | 0.08 | 0.1 | 0.01 | Low | stable |
| low1_0c | <0.01 | <0.01 | 0.97 | 0.08 | -0.01 | 0.01 | 0.55 | 0.02 | Low | stable |
| low1_0d | -0.09 | 0.08 | 0.13 | 0.06 | <0.01 | <0.01 | 0.95 | 0.02 | Low | stable |
| low1_0e | 0.13 | 0.13 | 0.04 | 0.06 | 0.02 | 0.07 | 0.14 | 0.01 | Low | stable |
| low10_0a | 0.01 | 0.02 | 0.49 | 0.02 | -0.02 | 0.03 | 0.29 | 0.02 | Low | stable |
| low10_0b | -0.02 | 0.04 | 0.28 | 0.02 | <0.01 | <0.01 | 0.9 | 0.02 | Low | stable |
| low10_0c | -0.06 | 0.16 | 0.02 | 0.02 | <0.01 | <0.01 | 0.89 | 0.02 | Low | stable |
| low10_0d | <0.01 | <0.01 | 0.9 | 0.04 | -0.02 | 0.03 | 0.32 | 0.02 | Low | stable |
| low10_0e | -0.04 | 0.08 | 0.13 | 0.02 | 0.01 | <0.01 | 0.7 | 0.02 | Low | stable |
| low20_0a | 0.04 | 0.07 | 0.16 | 0.02 | 0.03 | 0.07 | 0.14 | 0.02 | Low | stable |
| low20_0b | -0.06 | 0.14 | 0.04 | 0.03 | -0.01 | 0.01 | 0.63 | 0.02 | Low | stable |
| low20_0c | 0.04 | 0.08 | 0.11 | 0.02 | 0.04 | 0.11 | 0.06 | 0.02 | Low | stable |
| low20_0d | <0.01 | <0.01 | 0.92 | 0.02 | -0.04 | 0.09 | 0.08 | 0.02 | Low | stable |
| low20_0e | -0.04 | 0.1 | 0.08 | 0.02 | -0.01 | 0.01 | 0.65 | 0.02 | Low | stable |
| low5_0a | 0.06 | 0.12 | 0.06 | 0.03 | -0.01 | <0.01 | 0.71 | 0.02 | Low | stable |
| low5_0b | -0.04 | 0.06 | 0.19 | 0.03 | <0.01 | <0.01 | 0.78 | 0.01 | Low | stable |
| low5_0c | -0.03 | 0.02 | 0.44 | 0.04 | -0.01 | <0.01 | 0.71 | 0.02 | Low | stable |
| low5_0d | 0.01 | <0.01 | 0.85 | 0.04 | 0.01 | 0.01 | 0.59 | 0.02 | Low | stable |
| low5_0e | 0.02 | 0.02 | 0.42 | 0.03 | 0.02 | 0.03 | 0.34 | 0.02 | Low | stable |
| med1_0a | 0.04 | 0.02 | 0.39 | 0.05 | 0.02 | 0.06 | 0.17 | 0.01 | Medium | stable |
| med1_0b | -0.04 | 0.02 | 0.42 | 0.05 | <0.01 | <0.01 | 0.8 | 0.02 | Medium | stable |
| med1_0c | <0.01 | <0.01 | 0.94 | 0.05 | -0.02 | 0.06 | 0.14 | 0.01 | Medium | stable |
| med1_0d | 0.14 | 0.17 | 0.02 | 0.05 | -0.02 | 0.09 | 0.08 | 0.01 | Medium | stable |
| med1_0e | -0.04 | 0.03 | 0.32 | 0.04 | -0.01 | 0.02 | 0.37 | 0.02 | Medium | stable |
| med10_0a | 0.02 | 0.02 | 0.4 | 0.02 | -0.02 | 0.03 | 0.31 | 0.02 | Medium | stable |
| med10_0b | 0.02 | 0.01 | 0.53 | 0.03 | -0.01 | <0.01 | 0.75 | 0.02 | Medium | stable |
| med10_0c | -0.01 | 0.01 | 0.7 | 0.02 | -0.02 | 0.04 | 0.28 | 0.02 | Medium | stable |
| med10_0d | -0.03 | 0.04 | 0.29 | 0.03 | -0.02 | 0.05 | 0.22 | 0.02 | Medium | stable |
| med10_0e | -0.02 | 0.05 | 0.21 | 0.02 | -0.01 | 0.01 | 0.53 | 0.02 | Medium | stable |
| med20_0a | -0.03 | 0.03 | 0.36 | 0.03 | <0.01 | <0.01 | 0.9 | 0.03 | Medium | stable |
| med20_0b | 0.03 | 0.04 | 0.27 | 0.03 | -0.04 | 0.04 | 0.24 | 0.04 | Medium | stable |
| med20_0c | -0.01 | 0.01 | 0.6 | 0.03 | -0.04 | 0.05 | 0.21 | 0.03 | Medium | stable |
| med20_0d | <0.01 | <0.01 | 0.82 | 0.02 | 0.02 | 0.01 | 0.49 | 0.03 | Medium | stable |
| med20_0e | 0.01 | <0.01 | 0.72 | 0.02 | -0.05 | 0.06 | 0.15 | 0.03 | Medium | stable |
| med5_0a | 0.02 | 0.02 | 0.5 | 0.03 | -0.01 | 0.01 | 0.57 | 0.02 | Medium | stable |
| med5_0b | -0.03 | 0.07 | 0.14 | 0.02 | -0.02 | 0.05 | 0.19 | 0.02 | Medium | stable |
| med5_0c | -0.02 | 0.01 | 0.55 | 0.03 | -0.01 | 0.01 | 0.61 | 0.01 | Medium | stable |
| med5_0d | <0.01 | <0.01 | 0.96 | 0.03 | 0.02 | 0.04 | 0.26 | 0.02 | Medium | stable |
| med5_0e | -0.05 | 0.13 | 0.05 | 0.02 | -0.03 | 0.11 | 0.06 | 0.02 | Medium | stable |

Table S6. Summary table of the simulation exercise that includes the number of hunt areas that exhibited significant changes in horn size through time based on modeled horn size of 7-year olds and actual size of 7-year olds within the population for each combination of harvest rate (1, 5, 10, or 20 percent), selectivity (low, medium, or high), and direction (increasing, decreasing, or stable) in the simulated populations. We simulated 5 hunt areas for each combination of harvest rate, selectivity, and direction of change. Selectivity indicates the level of harvest selectivity that was imposed on a population (low – random harvest, medium – harvest randomly occurred from animals with horns larger than the 50% percentile, and high – harvest randomly occurred from animals with horns larger than the 75% percentile). Harvest rate indicates the percent of animals that were harvested from a population in each year. Increasing, Decreasing, and Stable indicate the direction of horn growth that occurred in the simulation over time. Predicted 7 Year Olds represents the number of hunt areas where trends were detected from modeled horn growth curves of all harvested males (identical approach to analyses within our manuscript). Population 7 Year Olds indicates the number of hunt areas where trends were detected in horn size of all 7-year olds within the population over time. All changes detected in simulated population with increasing and decreasing horn size where in the expected direction.

|  |  | | **Decreasing** | |  | **Increasing** | | |  | **Stable** | |
| --- | --- | --- | --- | --- | --- | --- | --- | --- | --- | --- | --- |
| **Selectivity** | | **Harvest Rate** | **Predicted 7 Year Olds** | **Population 7 Year Olds** |  | | **Predicted 7 Year Olds** | **Population 7 Year Olds** |  | **Predicted 7 Year Olds** | **Population 7 Year Olds** |
| Low | | 1 | 5 | 5 |  | | 5 | 5 |  | 1 | 0 |
| Low | | 5 | 5 | 5 |  | | 5 | 5 |  | 0 | 0 |
| Low | | 10 | 5 | 5 |  | | 5 | 5 |  | 1 | 0 |
| Low | | 20 | 5 | 5 |  | | 5 | 5 |  | 1 | 0 |
| Medium | | 1 | 5 | 5 |  | | 5 | 5 |  | 1 | 0 |
| Medium | | 5 | 5 | 5 |  | | 5 | 5 |  | 1 | 0 |
| Medium | | 10 | 5 | 5 |  | | 5 | 5 |  | 0 | 0 |
| Medium | | 20 | 5 | 5 |  | | 5 | 5 |  | 0 | 0 |
| High | | 1 | 5 | 5 |  | | 5 | 5 |  | 0 | 0 |
| High | | 5 | 5 | 5 |  | | 5 | 5 |  | 1 | 1 |
| High | | 10 | 5 | 5 |  | | 5 | 5 |  | 0 | 0 |
| High | | 20 | 5 | 5 |  | | 5 | 4 |  | 0 | 0 |
